# Cytotoxic and Radiosensitising Effects of a Novel Thioredoxin Reductase Inhibitor in Brain Cancers

**DOI:** 10.1007/s12035-022-02808-4

**Published:** 2022-03-28

**Authors:** Anqi Yao, Sarah J. Storr, Martyn Inman, Lucy Barwell, Christopher J. Moody, Stewart G. Martin

**Affiliations:** 1 Nottingham Breast Cancer Research Centre, School of Medicine, Biodiscovery Institute, University of Nottingham, University Park, Nottingham, NG7 2RD UK; 2 School of Chemistry, University of Nottingham, University Park, Nottingham, NG7 2RD UK

**Keywords:** Brain cancer, Epithelial-mesenchymal transition, Indolequinones, Radiation, Thioredoxin system

## Abstract

The thioredoxin (Trx) system, a key antioxidant pathway, represents an attractive target for cancer therapy. This study investigated the chemotherapeutic and radiosensitising effects of a novel Trx reductase (TrxR) inhibitor, IQ10, on brain cancer cells and the underlying mechanisms of action. Five brain cancer cell lines and a normal cell type were used. TrxR activity and expression were assessed by insulin reduction assay and Western blotting, respectively. IQ10 cytotoxicity was evaluated using growth curve, resazurin reduction and clonogenic assays. Radiosensitivity was examined using clonogenic assay. Reactive oxygen species levels were examined by flow cytometry and DNA damage assessed by immunofluorescence. Epithelial-mesenchymal transition (EMT)-related gene expression was examined by RT-PCR array. IQ10 significantly inhibited TrxR activity but did not affect Trx system protein expression in brain cancer cells. The drug exhibited potent anti-proliferative and cytotoxic effects against brain cancer cells under both normoxic and hypoxic conditions in both 2D and 3D systems, with IC_50_s in the low micromolar range. It was up to ~ 1000-fold more potent than temozolomide. IQ10 substantially sensitised various brain cancer cells to radiation, with such effect being due, in part, to functional inhibition of TrxR, making cells less able to deal with oxidative stress and leading to increased oxidative DNA damage. IQ10 significantly downregulated EMT-associated gene expression suggesting potential anti-invasive and antimetastatic properties. This study suggests that IQ10 is a potent anticancer agent and could be used as either a single agent or combined with radiation, to treat brain cancers.

**Supplementary Information:**

The online version contains supplementary material available at 10.1007/s12035-022-02808-4.

## Introduction

Brain tumours are the 9th most common cause of cancer deaths in the UK, with only 12% of patients surviving more than 5 years in England [1]. Amongst all malignant brain tumours, glioblastoma (GBM) is the most common and aggressive type in adults; whilst in children, the most prevalent type is medulloblastoma. Despite the use of multimodal treatment strategies, including surgery, radiation and chemotherapy, the prognosis of malignant brain tumours remains poor, particularly GBM, where the 5-year survival rate is only 5% [2]. Whilst survival rates for medulloblastoma patients are generally good, unfortunately relapses occur in around 30% of patients, and prognosis after recurrence is extremely poor, with a 5-year survival rate of < 10% [3, 4]. There is, therefore, an urgent need to develop novel targeted therapeutics.

Reactive oxygen species (ROS) are highly reactive chemicals formed from oxygen and are known to play a critical role in various physiological and pathological conditions. Under physiological conditions, intracellular ROS levels are under tight control by antioxidant systems; however, in pathological conditions, such as cancer, ROS production is increased [5] with such cells becoming increasingly reliant upon up-regulation of redox buffering systems to survive. A relative excess of ROS when compared with antioxidants is often defined as 'oxidative stress’ [6] and is one of the major ways by which conventional low linear energy transfer (LET) radiotherapy exerts its cytotoxic effects on cells [7]. As mentioned, redox homeostasis is often disrupted in cancer cells due to increased oxidative stress caused by accelerated cell proliferation, metabolism and growth [8]. As excessive generation of ROS can be toxic to cells, and with cancer cells that have buffering systems working at capacity, they are likely to be more sensitive to agents that increase ROS generation. Therefore, inducing ROS by exogenous agents whilst simultaneously inhibiting redox buffering capacity may represent a novel approach to selectively enhance cancer cell killing while simultaneously reducing normal tissue cytotoxicity [9].

The thioredoxin (Trx) system is a key cellular antioxidant pathway important in defence against oxidative stress and plays an important role in regulating therapeutic response of cancer cells [7]. The system comprises Trx reductase (TrxR), Trx and Trx-interacting protein (TxNIP) [10]. The expression levels of Trx/TrxR are often increased in a variety of human tumours and linked to tumour growth, progression, metastasis, treatment resistance and poor prognosis [11, 12]. A recent study by our group found that high TrxR expression in patient tumours was significantly associated with a worse prognosis in adult and paediatric gliomas and medulloblastomas [13]. Inhibiting the Trx system results in the modulation of the intracellular redox state, which can make cells more sensitive to certain chemotherapeutic agents and to radiation, through a variety of possible mechanisms including ROS accumulation and the alteration of certain signalling pathways [10, 14]. Trx system proteins have, therefore, emerged as promising biomarkers and drug targets for cancer therapy.

A number of TrxR inhibitors have been developed as potential anticancer agents or as adjuncts to existing therapies. The anti-arthritic drug auranofin has been shown to inhibit TrxR at nanomolar concentrations in ovarian cancer cells [15]. Auranofin was also shown to sensitise breast cancer stem cells to radiation [16], and the combination of auranofin, buthionine sulfoximine and radiation significantly increased the radioresponse and survival of breast cancer in in vivo models [17]. Polyphenols, such as curcumin, have also been shown to irreversibly inhibit TrxR and increase ROS production [18] and the radiosensitivity of prostate cancer cells [19]. Additionally, a study in human gliomas has demonstrated that tetrahydrocurcumin (a major metabolite of curcumin) exhibited promising radiosensitising potential both in vitro and in vivo [20]. Another TrxR inhibitor motexafin gadolinium (MGd) has been shown to enhance radiation responses in both animal models and clinical trials [21–23]. MGd appears to selectively concentrate in tumours to a greater extent than normal tissue, showing promise in a phase I clinical trial for patients with GBM [24]; however, in a later phase I/II trial, MGd in combination with radiotherapy and temozolomide (TMZ) did not result in a significant survival benefit compared to historical control [25]. Whilst the aforementioned agents target TrxR as a component of their mechanism of action, they also affect other targets.

We (M.I. and C.J.M.) have developed a series of antitumour TrxR inhibitors, namely indolequinones (IQs) [26, 27]. These agents inhibit TrxR activity at nanomolar concentrations and induced time- and concentration-dependent apoptosis in pancreatic cancer cell lines [27]. A subsequent study revealed that inhibition of TrxR by IQs led to Trx oxidation, dissociation of free ASK1, phosphorylation of JNK/p38 and subsequent apoptosis [28]. The mechanism of action of the IQs has been proposed to involve metabolic reduction by cellular reductases, loss of a leaving group to generate an electrophile resulting in alkylation of the selenocysteine residue in the active site of TrxR [27]. A recent study from our group reported that one of the IQs exhibited significant anticancer and radiosenstising effects in breast cancer cells [29]. The efficacy of IQs as single agents and the potential radiosensitising effect in brain cancer has not been previously examined. The current study investigates the single agent efficacy and radiosensitising properties of one of the most potent IQs, IQ10 [28], on a range of brain cancer cells and assesses potential mechanisms underlying its action.

## Materials and Methods

### Cell Culture

A panel of five different brain cancer cell lines (adult GBM cell line SNB19; paediatric GBM cell lines SF188 and KNS42; and medulloblastoma cell lines DAOY and UW228-3) and a normal cell type, MRC5 lung fibroblasts, were used in this study. Different cell lines were cultured in specific medium (Supplementary Table S8) at 5% CO_2_, 37 °C. Hypoxic incubations: 5% CO_2_ and 1% O_2_ at 37 °C in an INVIVO2400 workstation (Baker Ruskinn). Cells were authenticated approximately every 4–6 months using a multiplex short tandem repeat system (Powerplex® 16, Promega) and regularly tested for mycoplasma.

### Drugs

IQ10 was synthesised by Dr. Martyn Inman and Prof. Chris Moody (University of Nottingham, UK) according to a previously published procedure [27]. TMZ and auranofin were purchased from Sigma (UK) and chosen as control drugs. All drugs were dissolved in dimethyl sulfoxide (DMSO, Sigma) and kept aliquoted as stocks at − 20 °C.

### TrxR Activity Assay (Insulin Reduction)

Following incubation, with various concentrations of IQ10 or with 1 μM of auranofin, for 4/48 h, cells were trypsinised, counted and lysed in M-PER protein extraction reagent (Thermo Fisher) for 20 min. Lysates were analysed immediately or stored for future use at − 80 °C. The Bio-Rad protein assay kit (Bio-Rad Laboratories) was used to measure the protein concentration of each sample. TrxR activity was measured in 96-well plates using the endpoint insulin reduction assay as described previously [30] with appropriate modifications. Briefly, the assay mixture contained the following in a final volume of 100 μL HE buffer (100 mM HEPES, pH 7.2; 5 mM EDTA): 80 µg of protein, 2 µM *Escherichia coli* Trx, 1 mg/ml bovine insulin and 1 mM NADPH. Duplicate samples were used, either with or without exogenous Trx enzyme. A cell-free enzyme assay was simultaneously conducted, using 10 µL of different concentrations of TrxR (0, 0.025, 0.05, 0.1, 0.25, 0.5, 0.75 and 1 µM), to generate a standard curve. Reactions were incubated at 37 °C for 1 h and stopped by the addition of 150 µL of 10 mM DTNB, 6 M guanidine hydrochloride and 50 mMTris (pH 8.0). Absorbance was measured at 405 nm using a microplate reader (FLUOstar OPTIMA, BMG LABTECH). The absorbance of the sample lacking exogenous Trx was subtracted from that of the corresponding sample containing exogenous Trx. The difference in absorbance indicates TrxR function. The amount of functional TrxR was calculated from the standard curve, generated simultaneously, with pure enzymes. The TrxR activity was expressed as the percentage of DMSO-treated control.

### Western Blotting

Western blotting was performed as described previously [31]. Briefly, subconfluent cells were harvested and lysed in RIPA buffer (Sigma) supplemented with 1 × Halt™ Protease Inhibitor Cocktail (Thermo Fisher Scientific) on ice for 15 min, and then lysates were frozen at – 20 °C or – 80 °C for long-term storage. Lysates were loaded into a SDS-PAGE gel (Invitrogen^TM^Bolt™ 4–12% Bis–Tris Plus Gel), after which proteins were separated by gel electrophoresis and transferred onto a nitrocellulose membrane (GE Healthcare). Membranes were blocked with 5% (w/v) non-fat milk powder in 0.1% PBS/Tween 20 prior to incubation with primary antibody at 4 °C overnight. The primary antibodies used in this study were mouse anti-TrxR (1:1000, ab16847, Abcam), rabbit anti-Trx (1:5000, ab133524, Abcam) and rabbit anti-TxNIP (1:1000, ab188865, Abcam). Mouse anti-β-actin (1:2000, ab8226, Abcam) or rabbit anti-β-actin (1:1000, ab8227, Abcam) antibody was used as internal control. Membranes were washed and incubated with secondary antibodies [i.e. 680RD Donkey anti-Mouse IgG (926–68,072, IRDye, LI-COR) and 800CW Donkey anti-Rabbit IgG (926–32,213, IRDye, LI-COR)] for 1 h. Membranes were visualised using an Odyssey FC Imager (LI-COR Biosciences). The fluorescence intensity was quantified using Image Studio Version 4.1 (LI-COR Biosciences) and normalised against β-actin signals.

### Growth Assay

Cells (2 × 10^5^) were seeded in six-well plates, allowed to adhere overnight and then treated, in triplicate, with different concentrations of TMZ (0–1000 μM) or IQ10 (0–1 µM) for up to 72 h under normoxic or hypoxic conditions. DMSO was added to the negative control groups at the same volume equivalent to that in the treatment groups. For hypoxic experiments, cells were pre-incubated at 1% O_2_ for 24 h prior to drug treatment. Cells were then treated with different concentrations of IQ10 for up to an additional 72 h under hypoxia. Normoxic controls were always assessed in parallel to hypoxic-treated groups. Every 24 h after initiating drug treatment, cells were trypsinised and counted using a Countess system. Cell counts were recorded, analysed and plotted over the 72 h period. IC_50_ values were defined as the concentration of drug that resulted in a 50% reduction in cell number compared with DMSO-treated controls.

### Clonogenic Survival Assay

Cells collected from the growth assay (48 h time point) were counted and plated at low-density, in triplicate, and incubated at 37 °C, 5% CO_2_, undisturbed, for colony formation. After 12 days (21 days for KNS42), colonies were fixed (50% methanol in PBS), stained (0.5% crystal violet solution) and counted manually. Any cluster of cells greater than 50 in number was counted as a surviving colony. The plating efficiency (PE) was calculated as number of control colonies formed/number of control cells seeded. For single-agent treatment, drug or radiation, the surviving fraction (SF) was calculated as follows: number of colonies formed after treatment / (number of cells seeded × PE). For drug radiation combination experiments, cytotoxicity of drug treatment was accounted for by calculating SF as follows: number of colonies formed from each radiation dose / (number of cells seeded × PE × SF of drug-treated cell at 0 Gy).

### Spheroid Viability and Survival

Cells were seeded in ultra-low attachment round-bottom 96-well plates (Corning), aggregated by centrifugation (2000 rpm/10 min), and allowed to grow for 4 days under standard culture conditions to form mature spheroids. To compare the effects of IQ10 on cell viability between 3 and 2D cultures, cells were seeded in parallel in flat-bottom 96-well plates and cultured for 24 h. All samples were subsequently treated with different concentrations of IQ10 (0–1 µM) for 48 h. Viability was assessed using the resazurin reduction assay (CellTiter-Blue, Promega) following the manufacturer’s protocol. Briefly, medium in each well was replaced with a mixture of 100 μL fresh medium and 20 μL dye, and the plates were placed back in the incubator for 4 h. Fluorescence was measured with an excitation wavelength of 560 nm and emission 590 nm on a plate reader. Negative control cells were cultured with 0.01% DMSO. Six spheroids per condition were assessed in each experiment. The clonogenicity of cells cultured in spheroids was assessed using conventional 2D clonogenic survival assay as described above. Twelve replicate spheroids per condition were produced and left to grow for 4 days before being treated with IQ10 (0–1 µM) for an additional 48 h. 2D controls were plated the day before drug exposure and were treated at the same time as those in 3D culture. Afterwards, 12 spheroids from the same treatment conditions were pooled and dissociated with trypsin/EDTA for 10 min followed by mechanical dissociation through repeated pipetting. Trypsinisation was quenched using complete medium and cells plated using the conventional 2D clonogenic protocol. Cells from 2D controls were trypsinised and subjected to clonogenic assay in parallel.

### Cell Irradiation

Irradiation was conducted using an Xstrahl RS225 cabinet with a single dose of 2, 4, 6 or 8 Gy (Gy). X-rays were delivered at 195 kV, 10 mA at a dose rate of 0.87 Gy/min. The cabinet was fitted with a 0.5-mm copper filter and used at a 48.4-cm focus-to-skin distance. Sham-irradiated cells were used as controls. Cells/spheroids were treated with 1 µM of IQ10 for 4 h under normoxia prior to irradiation. For 2D hypoxic experiments, cells were treated with clonogenic IC_50_ doses of IQ10 for 48 h under 1% O_2_ and then irradiated. Cells/spheroids were then trypsinised and plated for clonogenic survival assay as described above. Survival curves were fitted to a linear-quadratic model using the software CS-CAL (German Cancer Research Centre). The mean values of parameters α, β, α/β and SF2 (surviving fraction at 2 Gy) were calculated from the fitted curves. The SER was calculated as the radiation dose needed for radiation alone divided by the dose needed for IQ10 plus radiation at a SF of 1%.

### TrxR siRNA

Cells were seeded, in triplicate, in 24-well plates, incubated overnight and transfected with either a pre-designed siRNA directed against human TrxR-1 (SI00050876, target sequence, 5′-CTGCAAGACTCTCGAAATTAT-3′, Qiagen) or mock-transfected with a scrambled siRNA (Allstars Negative Control siRNA, Qiagen). Briefly, 2.5 μL of 2 μM siRNA and 4.5 μL HiPerFect transfection reagent (Qiagen) were combined in 100 μL medium (serum and antibiotic free) and incubated at room temperature for 10 min to allow complex formation. Reaction mixtures were then added to 400 μL of fresh medium. Medium was aspirated from wells and replaced with the appropriate 500 μL of transfection solution. After 48 h of incubation, the cells were re-plated, allowed to adhere overnight and treated with various concentrations of IQ10 for an additional 48 h. Growth curve and/or clonogenic survival assays were used as the endpoint to assess the drug effects. Western blotting analysis of TrxR and β-actin (as a loading control) were used to confirm that TrxR expression was suppressed for the duration of the experiment.

### Intracellular ROS Levels

Intracellular ROS levels were measured as described previously [32]. Briefly, cells were treated with various concentrations of IQ10 for 4 h either alone or with subsequent exposure to 1 mM H_2_O_2_ (ROS positive control) for 1 h. Cells treated with DMSO, at the same dilution ratio as IQ10, were set as negative controls. Cells were then incubated with the cell-permeable dye 2′,7 ′-dichlorodihydrofluorescein diacetate (H_2_DCF-DA, Sigma) in fresh medium at a final concentration of 1 μM at 37 °C for 30 min. Cells were harvested and intracellular ROS levels assessed by measurement of fluorescence using a MACSquant flow cytometer (Miltenyi Biotec). A gate was set to include only healthy living cells in the analysis. Data were analysed using FlowJo 7.6.1 software (Tree Star) to obtain the median fluorescence intensity (MFI) of each group representing the amount of intracellular ROS generation.

### Immunofluorescence

Cells grown on BioCoat™ coverslips (Corning) were treated with DMSO or 1 µM IQ10 for 4 h prior to 2 Gy irradiation (sham irradiated cells as control). They were then fixed in 4% paraformaldehyde/PBS for 30 min, permeabilised and blocked in 0.3% Triton X-100 with 3% bovine serum albumin in PBS for another 30 min at room temperature. Cells were stained with anti-γH2AX antibody (1:500 dilution, Millipore) overnight at 4 °C followed by incubation with Alexa 647-conjugated goat anti-mouse secondary antibody (1:200 dilution, Abcam) for 1 h. Nuclei were counterstained with VECTASHIELD containing DAPI (Vector). Imaging was performed using a confocal microscope (Leica TCS SPE). The number of γH2AX foci was counted using ImageJ with at least 100 nuclei analysed for each condition.

### *RNA Extraction and Real-Time EMT PCR Array*

RNA extraction, EMT PCR array and data analysis were performed as described previously [31]. Briefly, subconfluent cells were treated with DMSO or 1 μM IQ10 for either 4 h or 24 h. Total RNA from treated cells was stabilised with RNA Protect Cell Reagent (Qiagen) and purified using the RNeasy Plus Mini Kit (Qiagen) according to the manufacturer’s instructions. RNA samples from three independent experiments were pooled together. After quantification using a NanoDrop spectrophotometer (Thermo Scientific), 500 ng of total RNA was reversed-transcribed into cDNA using a RT^2^ First Strand Kit (Qiagen) at 42 °C for 15 min followed by 95 °C for 5 min to stop the reaction. Real-time PCR was conducted using a human EMT RT^2^ Profiler™ PCR Array (PAHS-090Z, Qiagen) and RT^2^ SYBR Green Mastermixes (Qiagen). The PCR array profiles the expression of 84 key genes associated with the EMT process. The amplification conditions were as follows: 95 °C for 10 min, followed by 40 cycles of 95 °C for 15 s and 60 °C for 1 min. Samples were then run on an ABI-7500 Fast real-time PCR system (Applied Biosystems). Data analysis was performed using the online GeneGlobe Data Analysis Centre (https://www.qiagen.com/gb/shop/genes-and-pathways/data-analysis-center-overview-page/). Fold-change of target genes against the reference gene was calculated from 2^−ΔΔCt^ values. The C_T_ cut-off value was set at 35. Gene expression differences greater than twofold were considered as differentially transcribed.

### Statistical Analysis

Statistical analysis was carried out using SPSS 24.0 or GraphPad Prism 7 software. Data are expressed as mean ± standard deviation (SD) of at least three independent experiments unless otherwise stated. For comparing two variables, the Student’s *t* test was used, and one-way ANOVA was used to compare three or more groups. *P* values < 0.05 were considered statistically significant.

## Results

### IQ10 Effectively Inhibits TrxR Activity But Not Expression

**Fig. 1:**
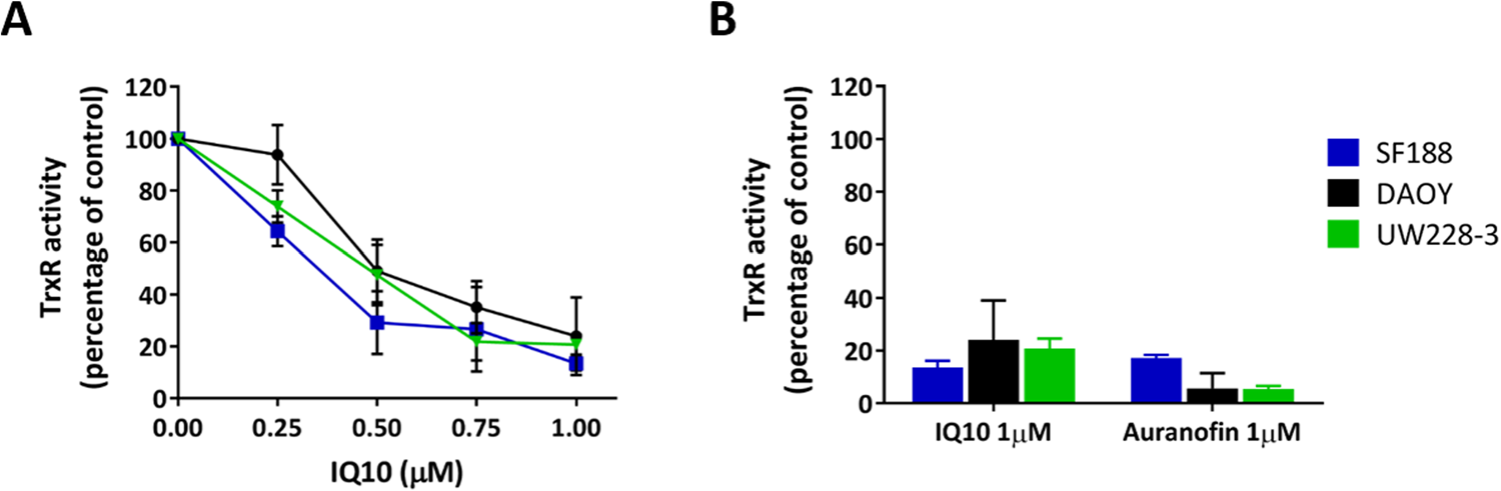
Effect of IQ10 on TrxR activity in brain cancer cells. Cells were treated with various concentrations of IQ10 or 1 μM auranofin for 4 h. TrxR activity was measured using the endpoint insulin reduction assay. **A** Inhibition of TrxR activity in SF188, DAOY and UW228-3 cells by IQ10. **B** Inhibition of TrxR activity in SF188, DAOY and UW228-3 cells by 1 μM of either IQ10 or auranofin. Data are expressed as a percentage of DMSO-treated control. Data represent the mean ± SD of three independent experiments, with each conducted in duplicate. Blue represents SF188, black represents DAOY, and green represents UW228-3

As illustrated in Fig. 1A, a 4-h IQ10 treatment significantly inhibited TrxR activity in a dose-dependent manner, with an IC_50_ of 342.74 ± 32.68 nM, 619.15 ± 43.54 nM and 443.26 ± 50.40 nM for SF188, DAOY and UW228-3 cells, respectively. At concentrations of 1 μM, the positive control drug auranofin caused an average TrxR inhibition of ~ 83–95% with IQ10 exhibiting similar potency, with an average inhibition of ~ 76–87% (Fig. 1B). The effect of IQ10 on expression of Trx system proteins was examined by Western blotting in DAOY and UW228-3 cells. In contrast to its inhibitory activity, IQ10 did not alter the expression of any of the Trx system proteins in either cell line either under normoxic or hypoxic conditions (Supplementary Fig. S1).

### Cytotoxicity of IQ10 on Brain Cancer Cells Under 2D Normoxic Culture

**Fig. 2:**
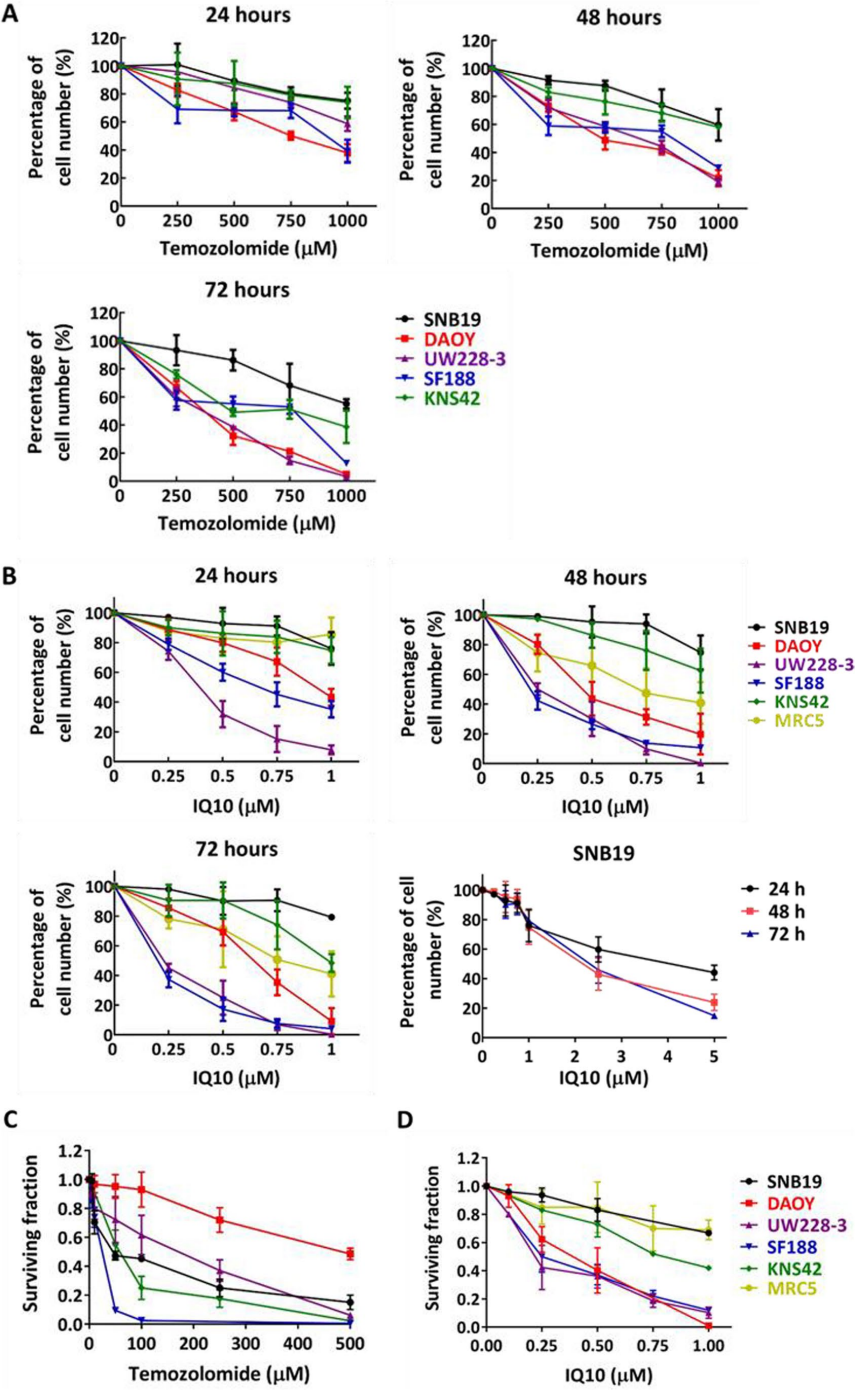
Cytotoxicity of TMZ and IQ10 under 2D normoxic conditions. A panel of five brain cancer cell lines and the MRC5 line were treated with various concentrations of TMZ (**A**) or IQ10 (**B**) for 24, 48 or 72 h. Total cell numbers in drug-treated cultures were plotted as a percentage of the vehicle control for each time point. SNB19 was treated with higher doses (up to 5 µM) of IQ10 to reach 50% growth inhibition. Data represent the mean ± SD of three independent experiments, with each experiment performed in triplicate. Cells (48-h time point) from growth assays were collected and plated for clonogenic survival assays (cells without drug treatment as control) (**C** and **D**). Data represent the mean ± SD of three independent experiments, with each experiment containing six parallel data sets. Plating efficiencies of individual cell lines were as follows: SNB19, 58 ± 9%; DAOY, 55 ± 10%; UW228-3, 92 ± 4%; SF188, 50 ± 9%; KNS42, 50 ± 6%; MRC5, 83 ± 8%. Black represents SNB19, red represents DAOY, purple represents UW228-3, blue represents SF188, green represents KNS42, and yellow represents MRC5

Cytotoxic effects of IQ10 were initially determined using a panel of five brain cancer cell lines and on MRC5 fibroblasts, under 2D normoxic conditions using growth curve and clonogenic survival assays. TMZ, a standard chemotherapeutic treatment for brain tumours, was used as a comparator and positive control. Growth curve data show a TMZ dose- and time-dependent inhibition of cell proliferation across all brain cancer cell lines (Fig. 2A), with IC_50_ values listed in Table 1. As shown in Fig. 2B, IQ10 inhibited cell proliferation at sub- or low micromolar concentrations, and also in a dose- and time-dependent manner. Amongst the five brain cancer cell lines, SF188 and UW228-3 were relatively more sensitive to IQ10, followed by DAOY, with SNB19 and KNS42 being the least sensitive. MRC5 cells were relatively resistant to IQ10 at the 24 h time point, but when the treatment time prolonged to 48 and 72 h, the response was comparable to the cancer lines. The IC_50_ values of IQ10 for each time point are listed in Table 1. In comparison to the IC_50_ values of TMZ, IQ10 was ~ 450–1000-fold more potent in terms of inhibiting cell proliferation. Clonogenic survival assays showed that 48 h treatment with either TMZ or IQ10 decreased cell survival in a dose-dependent manner (Fig. 2 C and D), suggesting a cytotoxic rather than cytostatic mechanism of action. The clonogenic IC_50_s of IQ10 were similar to the corresponding values from growth curve data (Supplementary Table S1) with MRC5’s being one of the most resistant cell types, alongside SNB19’s, suggesting that the drug may have the potential to preferentially kill cancer cells but spare normal cells. Further work is warranted. In comparison to TMZ, the clonogenic IC_50_s of IQ10 was ~ 30–1000-fold lower, indicating the increased potency of IQ10 over TMZ in terms of cell killing.

**Table 1:** Growth assay IC_50_ values of TMZ and IQ10

Cell lines	TMZ (μM)			IQ10 (μM)		
	24 h	48 h	72 h	24 h	48 h	72 h
SNB19	> 1000	> 1000	980.1 ± 280.8	3.8 ± 0.5	2.2 ± 0.3	2.0 ± 0.3
DAOY	546.9 ± 11.6	248.3 ± 49.8	162.5 ± 14.3	1.0 ± 0.2	0.5 ± 0.1	0.4 ± 0.1
UW228-3	> 1000	313.6 ± 17.8	148.4 ± 14.7	0.3 ± 0.1	0.2 ± 0.0	0.2 ± 0.0
SF188	> 1000	320.7 ± 86.8	188.0 ± 24.1	0.7 ± 0.1	0.2 ± 0.0	0.2 ± 0.0
KNS42	> 1000	> 1000	731.2 ± 24.3	> 1	> 1	1.0 ± 0.1
MRC5	NA	NA	NA	> 1	0.9 ± 0.2	0.8 ± 0.2

Data are expressed as mean ± SD of three independent experiments, with each experiment performed in triplicate. Abbreviations: *TMZ* temozolomide, *NA* not applicable/not performed

### Cytotoxicity of IQ10 Under 2D Hypoxic Culture and in 3D Spheroid Culture

**Fig. 3:**
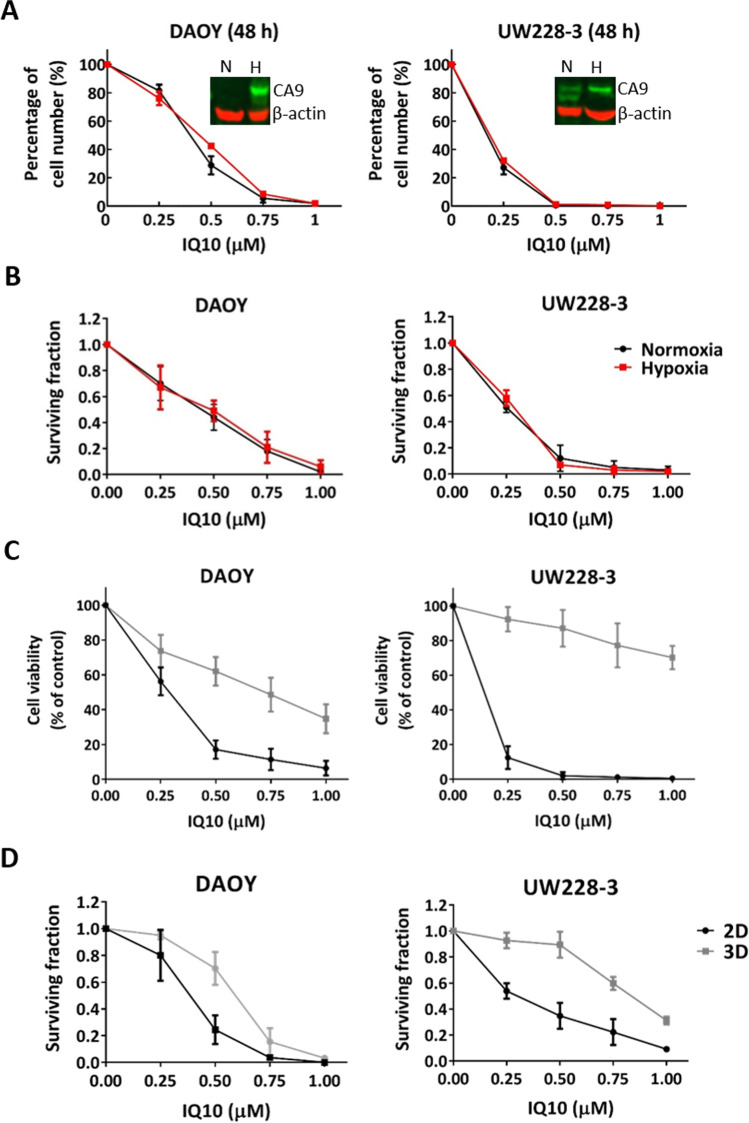
Cytotoxicity of IQ10 under 2D hypoxic conditions and in 3D spheroid culture. DAOY and UW228-3 cells were treated with various concentrations of IQ10 for 24, 48 and 72 h (cells treated with DMSO as control) under normoxia or hypoxia. **A** Cell number was plotted as a percentage of control for each time point shows the 48-h time point response. The data for 24- and 72-h time points are shown in Supplementary Fig. S2. Data represent the mean ± SD of three independent experiments, with each experiment performed in triplicate. The subpanels of the Western images show increased expression of hypoxia marker CA9 following 48 h of hypoxic incubation in DAOY and UW228-3 cells, confirming the induction of hypoxia. N, normoxia; H, hypoxia. Cells from the 48-h time point, from growth assays, were collected and plated out for clonogenic survival assays (cells treated with DMSO as control). **B** Data represent the mean ± SD of three independent experiments, with each experiment containing six parallel data sets. Plating efficiencies for normoxia and hypoxia were 55 ± 10% and 61 ± 11% for DAOY and 92 ± 4% and 81 ± 12% for UW228-3, respectively. Black represents normoxia and red represents hypoxia. **C** DAOY and UW228-3 cells, grown in 2D or as 3D spheroids, were treated with various concentrations (0–1 μM) of IQ10 for 48 h (cells treated with DMSO as control). Cell viability was determined by resazurin reduction assay. Percentage of cell viability normalised to DMSO-treated control is shown. Data represent the mean ± SD of three independent experiments, with each experiment performed in 6 replicate wells. **D** Clonogenic survival assays were performed to assess the surviving fraction of cells cultured in 2D or 3D spheroids. Data represent the mean ± SD of three independent experiments, with each experiment containing six parallel data sets. The average plating efficiencies for DAOY and UW228-3 cells in 2D were 72 ± 8% and 89 ± 5%; and plating efficiencies for spheroids were 62 ± 10% and 41 ± 8%, respectively. Grey represents 3D spheroids, and black represents 2D monolayer

Certain quinone-based agents (e.g. mitomycin C and apaziquone on which the IQs were based) show enhanced cytotoxic action under hypoxic conditions [33]. Thus, effects of IQ10 under hypoxia was assessed. In both DAOY and UW228-3 cells, IQ10 inhibited cell proliferation and clonogenicity in a time- and dose-dependent manner under both normoxic and hypoxic conditions, 1% O_2_ (Fig. 3 A and B; Supplementary Fig. S2). The corresponding IC_50_ values are listed in Supplementary Table S2. When comparing IC_50_s between the two culture conditions in both cell lines, the hypoxic cells appeared equally as sensitive as their normoxic equivalents to IQ10 at each time point, indicating that although there was no significant hypoxia-induced drug sensitisation, the potency of IQ10 was preserved in hypoxia.

Spheroid cultures are a useful model for understanding the influence of tumour-microenvironmental relationships on drug response. Resazurin cell viability assays showed that IQ10 was less effective toward DAOY and UW228-3 spheroids than their monolayers using a 48-h drug treatment (Fig. 3C). Consistent with such results, the clonogenic data also showed increased resistance to IQ10 in 3D spheroids (Fig. 3D). The IC_50_s of IQ10 in 2D vs. 3D cultures for both cell lines and the degree of resistance are listed in Supplementary Table S3. It is interesting to note that although both cell lines, particularly UW228-3, became more resistant to IQ10 when cultured in spheroids, the clonogenic IC_50_s were still less than 1 µM, indicating that IQ10 maintained its cytotoxic effect in 3D spheroid culture.

### IQ10 Sensitises Brain Cancer Cells to Irradiation in Normoxia, But Not in Hypoxia

**Fig. 4:**
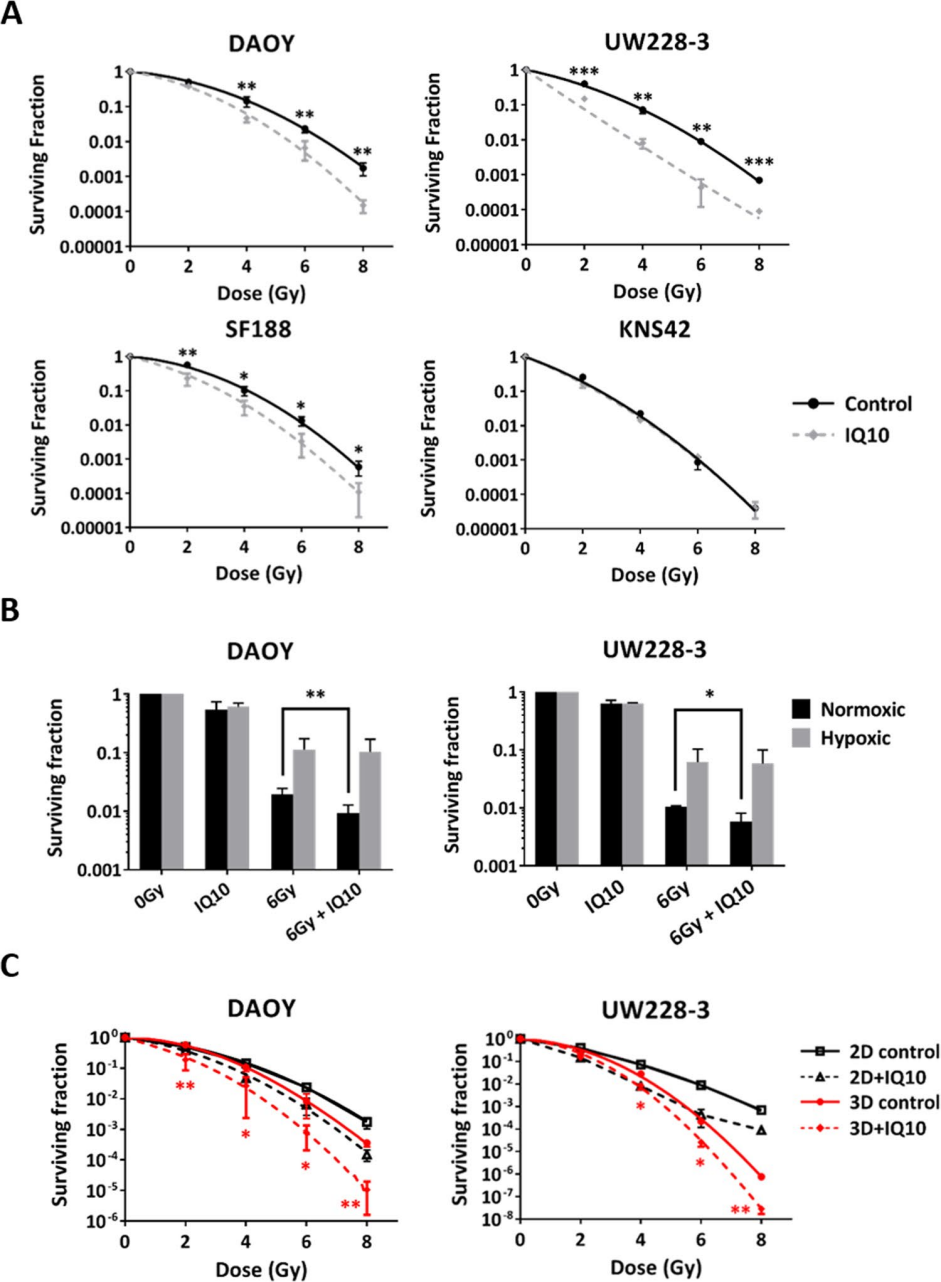
Effects of IQ10 on radioresponse of brain cancer cells in 2D (normoxia vs. hypoxia) and 3D cultures. **A** Survival curves for brain cancer cells exposed to single doses of irradiation with or without IQ10 treatment. DAOY, UW228-3, SF188 and KNS42 cells after IQ10 treatment (1 μM for 4 h under normoxia) were irradiated with single doses, and clonogenic survival assessed. Black represents radiation alone control, and grey represents radiation + IQ10. **B** Comparison of the radiosensitising effects of IQ10 on brain cancer cells between normoxia and hypoxia. DAOY and UW228-3 cells were treated with clonogenic IC_50_ doses of IQ10 for 48 h under either normoxic or hypoxic (1% O_2_) conditions. Cells were then irradiated with a single dose of 6 Gy and plated for clonogenic survival. Black represents normoxia and grey represents hypoxia. **C** Survival curves comparing the radiosensitising effects of IQ10 on DAOY and UW228-3 cells in 2D vs. 3D cultures. Plating efficiencies for DAOY, UW228-3, SF188 and KNS42 cells cultured in 2D normoxia were 63% (± 14%), 79% (± 16%), 81% (± 2%) and 64% (± 5%), respectively. Plating efficiencies for DAOY and UW228-3 cells cultured in hypoxia were 62% and 87% (± 10% and 6%), respectively. Plating efficiencies for DAOY and UW228-3 cells from 3D spheroids were 59% (± 5%) and 28% (± 7%), respectively. Data represent the mean ± SD of at least three independent experiments, with each experiment containing six parallel data sets. Statistical significance was determined by Student’s *t* test or one-way ANOVA and is indicated by asterisk. *P < 0.05, **P < 0.01 and ***P < 0.001. Black represents 2D monolayer, and red represents 3D spheroids

To determine whether modulating the TrxR activity by IQ10 could enhance the radiosensitivity of brain cancer cells, clonogenic survival assays were initially performed under 2D normoxic conditions. Results demonstrate that 4-h IQ10 treatment substantially increased radiosensitivity in all but the KNS42 cells with radiosensitisation most pronounced in UW228-3 (Fig. 4A). The sensitiser enhancement ratios (SERs) at 1% survival (SER_0.01_) were 1.25 ± 0.03, 1.68 ± 0.25, 1.22 ± 0.04 and 1.02 ± 0.02 for DAOY, UW228-3, SF188 and KNS42 cells, respectively (Table 2). The SF2s (surviving fraction at 2 Gy) of DAOY, UW228-3 and SF188 cells were significantly reduced by IQ10, further confirming the radiosensitising effect of IQ10 (*P* = 0.017, *P* = 0.001, and *P* = 0.048, respectively) (Table 2). Other changes in radiobiologic parameters including α, β, and α/β ratios are summarised in Table 2.

**Table 2:** Radiobiologic parameters of brain cancer cells treated with radiation ± IQ10 in 2D normoxic culture

Cell line	α (Gy^−1^)	β (Gy^−2^)	α/β (Gy)	SF2	SER_0.01_
DAOY					
IR alone	0.21 ± 0.07	0.07 ± 0.01	3.01 ± 1.28	0.50 ± 0.05	1.25 ± 0.03
IR + IQ10	0.34 ± 0.14	0.10 ± 0.02	3.81 ± 2.09	0.35 ± 0.07	
*t* test	*P* = 0.142	*P* = 0.059	*P* = 0.535	***P* = 0.017**	
UW228-3					
IR alone	0.40 ± 0.10	0.07 ± 0.01	6.58 ± 3.53	0.35 ± 0.05	1.68 ± 0.25
IR + IQ10	1.36 ± 0.28	0.00 ± 0.00	548.84 ± 65.54	0.07 ± 0.03	
*t* test	***P* = 0.005**	***P* = 0.002**	***P* < 0.001**	***P* = 0.001**	
SF188					
IR alone	0.15 ± 0.05	0.10 ± 0.00	1.56 ± 0.50	0.50 ± 0.05	1.22 ± 0.04
IR + IQ10	0.48 ± 0.27	0.08 ± 0.04	8.22 ± 8.39	0.29 ± 0.12	
*t* test	*P* = 0.111	*P* = 0.552	*P* = 0.242	***P* = 0.048**	
KNS42					
IR alone	0.69 ± 0.06	0.08 ± 0.00	9.25 ± 1.44	0.19 ± 0.02	1.02 ± 0.02
IR + IQ10	0.76 ± 0.05	0.07 ± 0.01	11.80 ± 1.97	0.17 ± 0.01	
*t* test	*P* = 0.212	*P* = 0.122	*P* = 0.143	*P* = 0.265	

Parameters were calculated from clonogenic data fitted to the linear-quadratic model. Data represent the mean ± SD of at least three independent experiments, with each containing six parallel data sets. Statistical significance was determined by Student’s *t* test. The *P* values are bold where they are ≤ 0.05. Abbreviations: *IR* irradiation, *SER*_0.01_ sensitiser enhancement ratio at 1% survival, *SF2* surviving fraction at 2 Gy

As tumour hypoxia is known to be radioprotective and a major contributor to therapeutic failure, the radiosensitising potential of IQ10 was further assessed under hypoxic conditions, 1% O_2_. Cells were treated with IQ10 under hypoxia for 48 h to ensure they were completely hypoxic prior to irradiation. The clonogenic survival data demonstrated that, as expected, hypoxia alone significantly increased radioresistance of both DAOY and UW228-3 cells (Supplementary Fig. S3). In line with the 4 h data (Fig. 4A), a 48-h IQ10 exposure under normoxic conditions also sensitised both cell lines to radiation (Fig. 4B); the SFs at 6 Gy were reduced from 1.96 to 0.93% (± 0.49% and 0.34%, *P* = 0.005) and from 1.05 to 0.58% (± 0.04% and 0.23%, *P* = 0.03) for DAOY and UW228-3, respectively. In contrast, no IQ10-induced radiosensitisation was observed under hypoxic conditions in either cell line (Fig. 4B). The results suggest that IQ10 is effective at enhancing radiosensitivity in normoxia, but such radiosensitising effects are not maintained in hypoxia. It should be noted, however, that the hypoxic radiosensitisation assessments were conducted after 48 h contact with IQ10 under hypoxia. Ideally, it would have been useful to assess the radiosensitising effect of IQ10 after a 4-h drug exposure at a concentration of 1 μM as these were the treatment conditions that already exhibited significant radiosensitising effect under 2D normoxic conditions. The lack of hypoxic radiosensitisation might be due to less efficient TrxR inhibition at 48 h.

### IQ10 Sensitises Brain Cancer Spheroids to Irradiation

As shown in Fig. 4C, both DAOY and UW228-3 cells in spheroids were, somewhat unexpectedly, significantly more sensitive to radiation than cells grown in 2D culture with effects more apparent at higher radiation doses. As with 2D culture conditions, IQ10 significantly enhanced the radioresponse of both DAOY and UW228-3 spheroids, with SER_0.01_ values of 1.38 ± 0.31 and 1.16 ± 0.01, respectively (Fig. 4C). When comparing the radiosensitising efficacy of IQ10 between 2 and 3D spheroid cultures (Fig. 4C), IQ10 seemed more effective in 3D spheroid culture than in 2D culture for DAOY cells (SER_3D_ = 1.38 ± 0.31 > SER_2D_ = 1.25 ± 0.03, *P* = 0.446), whereas IQ10 became less effective for UW228-3 spheroids (SER_3D_ = 1.16 ± 0.01 < SER_2D_ = 1.68 ± 0.25, *P* = 0.002) (Table 2; Supplementary Table S4). Collectively, irrespective of the system used (2D or 3D), it can be concluded that IQ10 sensitises both DAOY and UW228-3 cells to ionising radiation.

### Effects of TrxR Knockdown on Drug Response

**Fig. 5:**
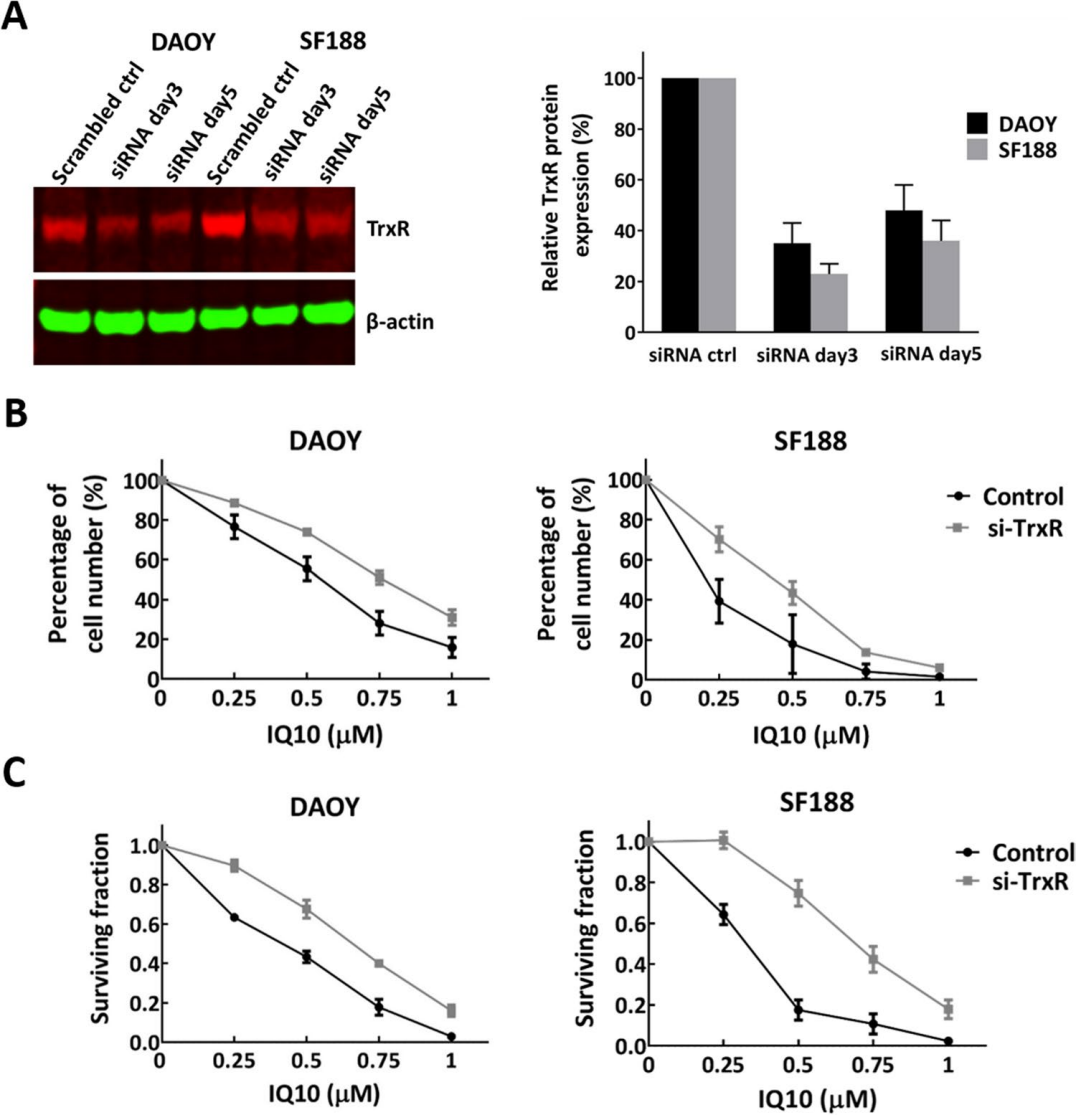
Effects of TrxR knockdown on the cytotoxicity of IQ10 in brain cancer cells. **A** Reduced TrxR expression after siRNA treatment in brain cancer cells. DAOY and SF188 cells were transiently transfected with 10 nM of TrxR siRNA or control scrambled siRNA. Transfection medium was removed after 48 h, replaced by fresh medium and incubated for an additional 72 h. Cells were collected at 3 or 5 days post-transfection and subjected to Western blotting to assess TrxR expression, with β-actin as an internal control. Data are expressed as an average percentage relative to the scrambled siRNA-treated control ± SD. Experiments were conducted three times, with separate cell lysates from different passage numbers of cells each time. Representative blots are shown. Black represents DAOY, and grey represents SF188. **B** Growth curve and **C** clonogenic survival assays were conducted to assess the cytotoxicity of IQ10 in siRNA transfected cells treated with various concentrations of IQ10 for 48 h. The plating efficiencies of cells were as follows: control DAOY, 56 ± 12%; si-TrxR DAOY, 65 ± 8%; control SF188, 52 ± 6%; si-TrxR SF188, 55 ± 11%. Data represent the mean ± SD of three independent experiments, with each experiment containing six parallel data sets. Black represents control cells, and grey represents TrxR siRNA transfected cells

To determine whether TrxR is an important, if not main, target of IQ10 a siRNA approach was used to knockdown TrxR expression in both DAOY and SF188 cells. Western blotting analysis demonstrated that TrxR expression was reduced to ~ 23–48% of the controls for the duration of the drug treatment (Fig. 5A). The cell growth and survival data (Fig. 5 B and C) showed that TrxR knockdown cells were still sensitive to IQ10 but that there was a marked reduction in cytotoxicity accompanied with a 1.5- to 2.2-fold increase in IC_50_s (Supplementary Table S5), suggesting TrxR as the drug target.

### IQ10 Elevates Intracellular ROS Production

**Fig. 6:**
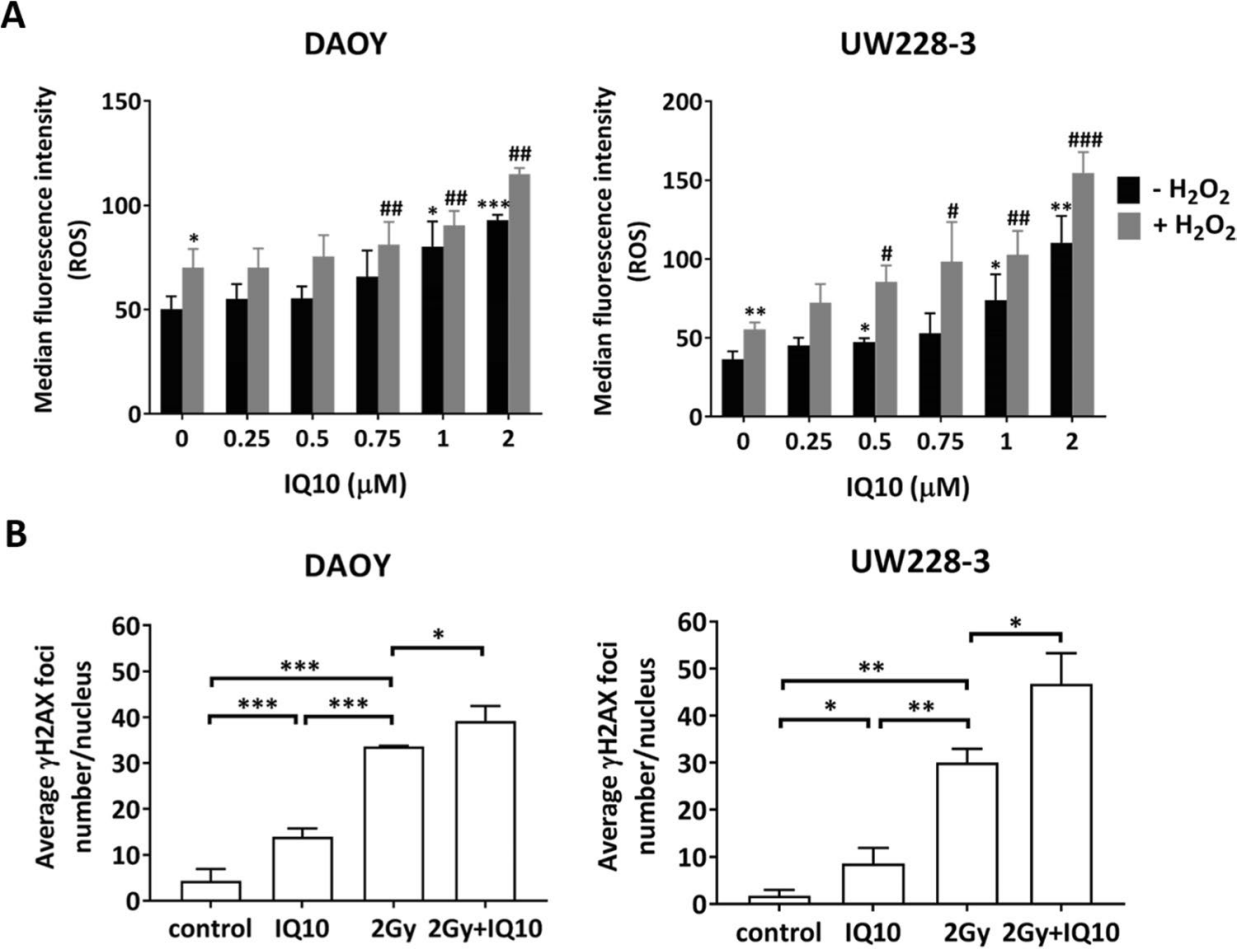
Effect of IQ10 on ROS levels and DNA damage. **A** Flow cytometry analysis of intracellular ROS levels of DAOY and UW228-3 cells treated with varying concentrations of IQ10 for 4 h with or without subsequent 1 mM of H_2_O_2_ for 1 h (cells without treatment as control). Median fluorescence intensity (MFI) was calculated and plotted. Data represent the mean ± SD of three independent experiments, with each conducted in triplicate. *P < 0.05, **P < 0.01, ***P < 0.001, IQ10/ H_2_O_2_ vs. untreated control. #*P* < 0.05, ##*P* < 0.01, ###*P* < 0.001, IQ10 + H_2_O_2_ vs. H_2_O_2_ alone. Black represents cells treated with IQ10 alone, and grey represents cells treated with IQ10 + subsequent H_2_O_2_. **B** Quantification of γH2AX foci in DAOY and UW228-3 cells following treatment with IQ10 and/or radiation. Cells were treated with or without IQ10 (1 µM) for 4 h prior to irradiation (2 Gy). DNA DSBs were analysed by confocal microscopy using the γH2AX foci measurements. Figures show the average number of γH2AX foci/nucleus, with more than 100 cells analysed each time. Data represent the mean ± SD of three independent experiments, with each conducted in triplicate. *P < 0.05, **P < 0.01, ***P < 0.001. Statistical significance was determined by one-way ANOVA

To help elucidate potential mechanisms responsible for the enhanced radiosensitisation by IQ10, intracellular ROS levels were examined by flow cytometry. Both cell lines responded similarly to the positive control, H_2_O_2_, with ROS levels increasing to 1.4-fold and 1.5-fold compared to control in DAOY (*P* = 0.01) and UW228-3 (*P* = 0.008), respectively. IQ10 ± H_2_O_2_ treatment increased intracellular ROS levels in a dose-dependent manner in both cell lines (Fig. 6A). At 2 μM IQ10 ± H_2_O_2_, intracellular ROS levels were elevated to 1.8 (IQ10 alone) and 2.2-fold (IQ10 + H_2_O_2_) of control in DAOY (*P* < 0.001 and *P* = 0.003, respectively). The effect of IQ10 on ROS generation was more pronounced in UW228-3 cells, with the same treatments resulting in a 3.0- and 4.3-fold increase compared to control (*P* = 0.002 and *P* < 0.001). Such data suggest that IQ10 significantly impairs cellular ability to cope with oxidative stress.

### IQ10 Increases γH2AX Formation Alone and in Response to Radiation

It was of interest to assess if IQ10-induced ROS elevation could lead to DNA damage, particularly double-strand breaks (DSBs). Spontaneous DNA DSBs were found in untreated control cells (~ 2–4 γH2AX foci/nucleus) (Fig. 6B; Supplementary Fig. S4). γH2AX foci generation was significantly increased in both cell lines as compared to the controls following IQ10 or radiation treatment, with the increase significantly higher in the 2 Gy irradiated group than in IQ10 treatment group (*P* < 0.01). When combining IQ10 with radiation, the number of γH2AX foci was increased to an even greater extent than achieved by irradiation alone. The average γH2AX foci number was increased from 33.6 ± 0.13 in irradiation alone group to 39.2 ± 3.3 in combined group for DAOY (*P* = 0.01) and from 30.0 ± 3.0 to 46.8 ± 6.5 for UW228-3 (*P* = 0.04) (Fig. 6B). Such results suggest that IQ10 treatment enhances formation of DNA DSBs, either directly or indirectly via ROS induction, and enhances the amount of DNA damage induced by radiation.

### Effect of IQ10 on EMT-Related Gene Expression

TrxR may also be involved in regulation of cell migration and invasion. RT^2^ Profiler PCR Arrays were used to investigate epithelial-mesenchymal transition (EMT)-related gene expression profiles of DAOY and UW228-3 cells treated by IQ10. Gene expression patterns were first compared in vehicle control DAOY and UW228-3 cells at 4 h/24 h time points. When comparing UW228-3 with DAOY, a total of 47 genes were differentially expressed. MMP3 and SPP1 genes were shown to be particularly differentially expressed (MMP3: 167.11-fold at 4 h and 57.41-fold at 24 h; SPP1: 61.01-fold and 44.92-fold, at 4 h and 24 h, respectively), whereas CAV2 and IGFBP4 genes were expressed at low levels (CAV2: − 7022.89-fold at 4 h and − 5153.72-fold at 24 h; IGFBP4: − 11,539.35-fold and − 7702.94-fold, at 4 h and 24 h, respectively). Other differently expressed genes are listed in Supplementary Table S6. The respective vehicle control group expression data were compared against the IQ10-treated group at each time point in each cell line. A full list of the differentially expressed (≥ twofold) EMT-related genes is presented in Supplementary Table S7. At 4-h, IQ10 significantly repressed expression of 18 genes in the DAOY cell line, inducing expression of only 2 genes, whilst the largest changes in gene expression were observed with KRT19 (9.49 fold) and SNAI3 (− 5.75 fold). At the 24-h time point, IQ10 significantly downregulated 17 genes and upregulated 3 genes with ERBB3 (5.94-fold) and MST1R (-69.07-fold) showing the largest changes in expression. In UW228-3 cells, IQ10 significantly repressed expression of 14 genes at the 4-h time point, inducing expression of 8 genes. The largest changes in gene expression were seen with SNAI3 (4.72 fold) and KRT19 (− 4.38 fold). At the 24-h time point, IQ10 significantly downregulated expression of 39 genes; upregulating expression of only 5 genes, PLEK2 (7.01 fold) and KRT19 (− 18.77 fold) had the largest changes in gene expression. Such data suggest that IQ10 may play an important role in regulating the expression of a variety of genes involved in EMT, suggesting that IQ10 treatment may be able to decrease EMT and influence EMT-associated effects on response, invasion and migration.

## Discussion

IQs were previously found to be potent inhibitors of TrxR activity in pancreatic cancer cells and cell-free systems [27]. Current data confirm IQ10 as a potent TrxR inhibitor with a dose-dependent decrease in activity in brain cancer models and with inhibition equivalent to the well-characterised TrxR inhibitor auranofin. The role of IQ10 in regulating expression of Trx family proteins was also assessed; however, no significant alterations in TrxR expression were observed. Such data are consistent with the study conducted by Yan and colleagues [27], which also found that total TrxR protein levels were unaltered following IQ treatment in pancreatic cancer cells. However, a dose-dependent decrease in the amount of free selenocysteine TrxR was observed and treatment with either IQ1 or IQ2 induced a dose-dependent increase in oxidised Trx expression accompanied by a decrease in the reduced form [28]. Although current data show no alterations in the expression of Trx system proteins, further experiments might wish to assess the expression of free selenocysteine TrxR and oxidised/reduced Trx after IQ10 treatment. Taken together, results from the current study suggest that IQ10 did not alter TrxR expression, but significantly inhibited its functional activity in brain cancer cells. The decreased TrxR activity in cells may be attributed to the direct inhibition of the enzyme activity by IQ10 rather the indirect alteration of protein expression.

The cytotoxicity of IQ10 was assessed in both normoxic and hypoxic conditions and in 2D vs. 3D cultures. Results demonstrate that IQ10 substantially decreases proliferation and clonogenic survival of brain cancer cells under both normoxic and hypoxic conditions with comparable IC_50_s, in the sub- to low micromolar range. In comparison to TMZ, a standard chemotherapeutic treatment for brain tumours, IQ10 was up to ~ 1000-fold more potent. Yan and colleagues have shown potent cytotoxic activity in which they found that IQs displayed potent cytotoxicity against pancreatic cancer cell lines with growth inhibitory IC_50_s in the low nanomolar range [27, 28]. Screening of selected IQs using the NCI-60 panel suggested particular effectiveness in colon, renal and melanoma cancers [27, 28]. In order to investigate whether IQ10 can exert toxic effect on cancer cells while maintaining a low toxicity to normal cells, MRC5 fibroblast cells were included in the cytotoxicity study. The results demonstrated that IQ10 seemed less toxic to MRC5 cells than to the majority of brain cancer cell lines tested. However it would be interesting to assess the cytotoxic and radiosensitising effects of IQ10 on a normal brain cell line (e.g. normal human astrocytes). As patients with brain tumours are particularly at risk for adverse late brain effects after (chemo)-radiotherapy, further experiments are required to explore the neurotoxic effects of IQ10, with and without radiation, on at least one normal brain cell line.

In brain tumours, especially GBM, hypoxia is strongly linked to tumour progression, chemoradiotherapy resistance and poor patient outcomes [34]. Hence, agents capable of overcoming hypoxic resistance would be beneficial for brain tumour treatment. As TrxR has been reported to be upregulated during hypoxia [35] and as the IQs were developed from agents that required bioreduction for full activity (i.e. apaziquone [36] and mitomycin [37]), it was of interest to know whether IQ10 would be more effective under hypoxic conditions. No increased hypoxic cytotoxicity was, however, observed suggesting involvement of other reductases or activation mechanisms. This lack of requirement for hypoxic bioactivation makes the compound potentially more amenable for clinical use as biological half-life and requirement for hypoxic bioactivation of previous generation agents have somewhat limited their clinical utility [38]. Although hypoxia has been shown, with certain agents (e.g. cisplatin, doxorubicin and etoposide) [39], to induce chemoresistance, no such resistance was evident in the current study.

The traditional 2D cell culture model, used in the current study, cannot fully mimic the in vivo cellular microenvironments with conclusions made from such 2D models requiring careful interpretation. 3D spheroid culture models have been developed and are being increasingly utilised in preclinical evaluation of novel anticancer agents [40]. The current study used a 3D spheroid model to compare the efficacy of IQ10 on brain cancer cells in parallel 2D and 3D experiments. Compared with 2D data, brain cancer cells cultured as 3D spheroids were more resistant to IQ10. Consistent with such data, a number of studies have also found that cells cultured in 3D systems are more refractory to anticancer agents than cells grown in 2D cultures [41–44]. Such changes in drug sensitivity between 2 and 3D culture models are likely to be driven by various factors, including the cell–cell interactions, signalling pathway activations, cellular microenvironment and also drug uptake rate [45–47]. The differential effects and increased 3D resistance does not seem to be related to differences in drug uptake as immunocytofluorescence studies show robust perfusion throughout spheroids (data not shown).

It has been reported that TrxR is often overexpressed in many aggressive cancers and that inhibition of TrxR can sensitise cancer cells to radiation [48–50]. We have shown that TrxR is expressed in both adult and paediatric brain tumours with high expression correlating with a worse prognosis [13]. Although not used in routine clinical treatments, TrxR inhibitors such as MGd [51, 52], auranofin [17] and curcumin [53] have been shown to act as promising radiosensitisers in the treatment of various cancer types. Clonogenic survival data from the current study demonstrate that, in normoxic conditions, IQ10 significantly increases radiosensitivity in all but the KNS42 cell model. The somewhat aberrant KNS42 paediatric GBM cell line data may be a reflection of the experiment using a uniform, and rather low, concentration of IQ10 (1 µM), that may not be sufficient to induce radiosensitivity in this line — use of equitoxic doses would be of interest in the future. In addition, intrinsic radiosensitivity might also affect IQ10 radiosensitisation as the agent appeared to be more effective in radioresistant than in radiosensitive lines. KNS42, as the most radiosensitive line in this study, may be more difficult to radiosensitise as it is already very sensitive to the killing effects of ionising radiation.

Whilst no increased single agent hypoxic sensitisation was observed with IQ10, hypoxic cancer cells are well recognised to be more resistant to radiotherapy and to represent the most aggressive fraction of a tumour. Preliminary experiments were conducted to evaluate the potential of IQ10 as a 2D hypoxic radiosensitiser. Surprisingly, no hypoxic radiosensitisation was observed, and this might be due to decreased TrxR inhibition at 48 h compared to 4 h (Supplementary Fig. S5). Therefore, short exposure is essential as the drug half-life may be insufficient to maintain long term enzyme inhibition — with further work required to determine biological stability and PK/PD parameters.

As with the single agent drug studies (above), the 3D spheroid model was used to investigate and compare the radiosensitivity in 2D vs. 3D in vitro cultures. Unlike the single agent drug response, cells cultured as spheroids became more sensitive to irradiation than those grown as 2D monolayers — this finding is in contrast to others who report certain cancer cells being generally more radioresistant in 3D culture [54]. The reason for these divergent results remains unclear. Possible explanations could include different methodological approaches and/or individual characteristics of the cell lines. The radiosensitising effects of IQ10 were also evaluated using the 3D spheroid model. Consistent with the 2D data, IQ10 also sensitised brain cancer spheroids to irradiation, indicating that the hypoxic fraction in the spheroids did not unduly affect sensitisation and further suggesting that IQ10 may be a valuable radiosensitiser candidate for brain cancer treatment.

To explore the potential mechanisms underlying the cytotoxic effects of IQ10, a siRNA approach was used to knockdown TrxR expression, with results showing that siRNA transfection reduced majority of TrxR expression and the TrxR-silenced cells became less sensitive to IQ10 treatment. Such results suggest that the cytotoxicity of IQ10 is due, in part, to functional inhibition of TrxR and that TrxR is an important target for IQ10. However, other potential targets may also be involved in IQ10’s anticancer activity. TrxR expression was unable to be completely and permanently inhibited by the siRNA approach; therefore, it is unclear how residual TrxR activity might affect the cytotoxicity of IQ10 in transfected cells. More robust models, perhaps using shRNA or CRISPR systems, are required to further validate the siRNA results.

Flow cytometry data show that IQ10 treatment elevates intracellular ROS levels with a further increase evident when combined with H_2_O_2_, suggesting that cells treated with IQ10 are less able to deal with induced oxidative stress. In line with this, IQ10 treatment increased the amount of radiation-induced DNA damage. Taken together, current results suggest that IQ10 inhibits TrxR activity, decreasing ROS scavaging ability, leading to increased intracellular ROS accumulation and subsequent induction of oxidative DNA damage, ultimately allowing the radiosensitisation of brain cancer cells when oxidative stress is increased even further. Additional mechanisms may also operate but require further study.

GBMs are frequently invasive and medulloblastomas, especially group 3 and 4, are often metastatic, resulting in poor patient prognosis. EMT is a key process in cancer progression and metastasis, making its inhibition an attractive therapeutic strategy [55]. EMT has been largely studied in various types of carcinomas (epithelial origin) but much less is known in nonepithelial tumour types such as brain tumours [56]. Several EMT-related transcription factors have been shown to play critical roles in the mesenchymal transformation of GBM including SNAI1/2 and Twist-1 [57]. It has been reported that SHH activation in medulloblastoma cells induces the expression of SNAI1, consequently activating the proto-oncogene N-MYC to induce cellular proliferation and transformation [58]. Although not extensively studied as yet, EMT appears to play an important role in brain tumours. A recent study, from our group, suggests that the overexpression of TrxR is associated with poor prognosis of brain tumour patients. The role of TrxR inhibition in the regulation of EMT was assessed by expression profiling as alterations to this process may be associated with the decreased overall survival seen in the patient samples [13]. EMT array results revealed that TrxR inhibition by IQ10 markedly downregulated a large number of EMT-related genes in brain cancer cells, including several master EMT effectors N-cadherin, Snail, Twist, Vimentin and Zeb, which are usually increased during EMT [59]. To our knowledge, few studies have been conducted to explore the relevance between the Trx system and EMT in brain cancers. This study identified, for the first time, that targeting TrxR by IQ10 might inhibit the migration and invasion of brain cancer cells through inhibiting pivotal EMT-related genes.

Poor drug penetration across the blood brain barrier (BBB) is one of the largest barriers to effective treatments in brain tumours [60]. Therefore, determination of drug’s BBB permeability is a prerequisite for screening drugs which could take effects in CNS [61]. For molecules to penetrate the BBB, a polar surface area (PSA) < 90 and a molecular weight < 500 Da are usually needed [62, 63]. With a PSA of 68.31 and a molecular weight of 450.41 Da, IQ10 may have the potential to cross the BBB. As such, preliminary experiments were conducted to predict the BBB penetration ability of IQ10 using caco-2 in vitro system with preliminary data suggesting that IQ10 was reasonably permeable and was not a substrate for efflux transporters (data not shown). A further study to assess the BBB penetration of IQ10 in male CD-1 mice is ongoing; with such data generated in the future, the BBB penetration ability of IQ10 will be better understood. If BBB penetration limits drug delivery, there are other potential delivery strategies that could be considered, such as direct intratumoural delivery via convection enhanced delivery, nanoparticle delivery or intracerebral implants, intrathecal delivery and hydrogel-based delivery [64, 65].

All results in this study were obtained using established in vitro cell line models; therefore, additional studies may wish to consider using primary, patient derived cells, tumour xenografts or ideally orthotopic murine models to confirm the current findings and to further link experimental studies with the clinical setting. More importantly, the pharmacokinetic (PK, concentration vs. time) and pharmacodynamic (PD, effect vs. time) parameters of IQ10 should be examined in animal models, as PK/PD profiles are crucial for the prediction of drug efficacy in humans. In addition, the PK/PD data could help guide subsequent study design of in vivo drug-radiation combination and fractionation experiments.

The Trx system is a key antioxidant pathway in defence against oxidative stress. Protein expression of Trx system family members has been linked to various clinicopathological variables, disease progression, treatment response and survival outcomes in many cancers, including brain tumours [13]. Expression of TxNIP, a member of the Trx system, is regulated by a number of factors and known to be targeted by one or more MicroRNAs (miRNAs) [66, 67]. MiRNAs are a class of small single-stranded non-coding RNAs (containing about 18–25 nucleotides) that function in RNA silencing and posttranscriptional regulation of gene expression [68]. They participate in numerous biological functions such as cell proliferation, differentiation and apoptosis. Evidence has also revealed that miRNAs are deregulated in various human cancers, including brain cancers [69, 70]. For example, miR-129–2 was downregulated in glioma tumours and cell lines, with enforced expression of miR-129–2 repressing glioma cell growth, migration and invasion and promoting cell apoptosis in vitro [69]. In GBM tissues, miR-127-3p was downregulated compared with normal brain tissues and miR-127-3p overexpression inhibited GBM cell growth by inducing G1-phase arrest both in vitro and in vivo [71]. It would be interesting to examine miR expression following TrxR inhibition by IQ10 treatment and to possibly explore the potential of using miR-127-3p and miR-129–2 as targets for brain tumour therapy.

Overall, the current study shows that IQ10 is a very potent TrxR inhibitor, exhibiting single agent anti-proliferative and cytotoxic effects under both normoxic and hypoxic conditions, in a variety of both 2D and 3D brain cancer cell line models. IQ10 is up to 1000-fold more potent than TMZ, the agent currently used clinically to treat brain tumours and used as a comparator in this study. IQ10 seems to preferentially kill brain cancer cells but spare normal fibroblasts MRC5. TrxR is confirmed as an important target of IQ10 and inhibition of it by IQ10 substantially sensitises both 2D and 3D cultured brain tumour cells to radiation, with this radiosensitising effect due, in part, to functional inhibition of TrxR activity, making cells less able to deal with oxidative stress and leading to increased oxidative DNA damage and cell killing. In addition, IQ10 might be a potential anti-migratory agent as treatment significantly downregulated EMT-related gene expression. Collectively, the current study highlights IQ10 as a promising agent, delivered either singly or combined with radiation, to improve outcome in brain tumours.

## Supplementary Information

Below is the link to the electronic supplementary material.

Supplementary file1 (DOCX 1480 KB)

## Data Availability

All data are available in the manuscript and its supplementary information files.
