# Supplementary material for: Cytotoxic and Radiosensitising Effects of a Novel Thioredoxin Reductase Inhibitor in Brain Cancers
Source: Mol Neurobiol. 2022 Mar 28;59(6):3546–63. doi: 10.1007/s12035-022-02808-4 (PMC9148287; doi:10.1007/s12035-022-02808-4)
Supplement: Supplementary file 1 — Supplementary file1 (DOCX 1480 KB) [file 12035_2022_2808_MOESM1_ESM.docx]

**Cytotoxic and radiosensitising effects of a novel thioredoxin reductase inhibitor in brain cancers**

Anqi Yao^1^, Sarah J. Storr^1^, Martyn Inman^2^, Lucy Barwell^1^, Christopher J. Moody^2^, Stewart G. Martin^1^

1 Nottingham Breast Cancer Research Centre, Biodiscovery Institute, School of Medicine, University of Nottingham, University Park, Nottingham NG7 2RD, UK

2 School of Chemistry, University of Nottingham, University Park, Nottingham NG7 2RD, UK

Correspondence should be addressed to Stewart G. Martin ([stewart.martin@nottingham.ac.uk](mailto:stewart.martin@nottingham.ac.uk)).

Address: Nottingham Breast Cancer Research Centre, Biodiscovery Institute (C209b), University of Nottingham, University Park, Nottingham NG7 2RD, UK

**Supplementary Material**

**Table of Contents**

**- Fig. S1**

**- Fig. S2**

**- Fig. S3**

**- Fig. S4**

**- Fig. S5**

**- Table S1**

**- Table S2**

**- Table S3**

**- Table S4**

**- Table S5**

**- Table S6**

**- Table S7**

**- Table S8**

**Fig. S1 Effect of IQ10 on Trx system protein expression in DAOY and UW228-3 cells under normoxic or hypoxic conditions.** Cells were treated with IQ10 (IC_50_ dose) for 48 h in normoxia or hypoxia, then harvested and subjected to Western blotting to assess the expression of Trx system proteins with β-actin as an internal control. (A) Representative blots of three independent experiments.(B) Bar charts represent the mean ± SD of three independent experiments, with separate cell lysates from different passage numbers of cells. The endogenous TxNIP expression was significantly lower, in DAOY than in UW228-3, while the TrxR and Trx expression were the same in the two cell lines. ****P*<0.001 (Student’s *t*-test). Abbreviations: M, marker; Trx, thioredoxin; TrxR, thiredoxinreductase; TxNIP, thioredoxin-interacting protein.


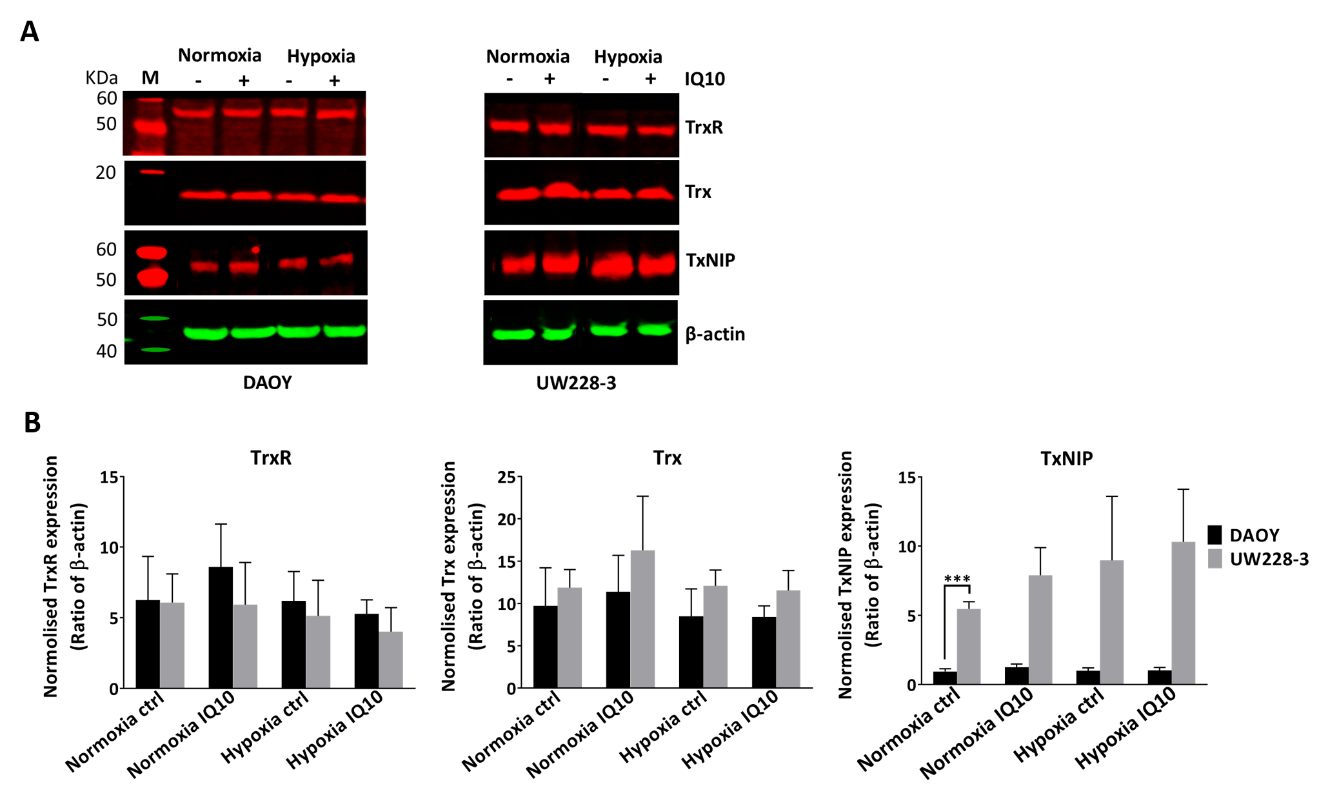


**Fig. S2 Effect of IQ10 on cell proliferation of brain cancer cells under normoxic or hypoxic conditions.** DAOY and UW228-3 cells were treated with various concentrations of IQ10 for 24, 48, and 72 h (cells without IQ10 treatment as control) under normoxia or hypoxia. Cell number was plotted as a percentage of control for each time point. Data represent the mean ± SD of three independent experiments, with each experiment performed in triplicate.


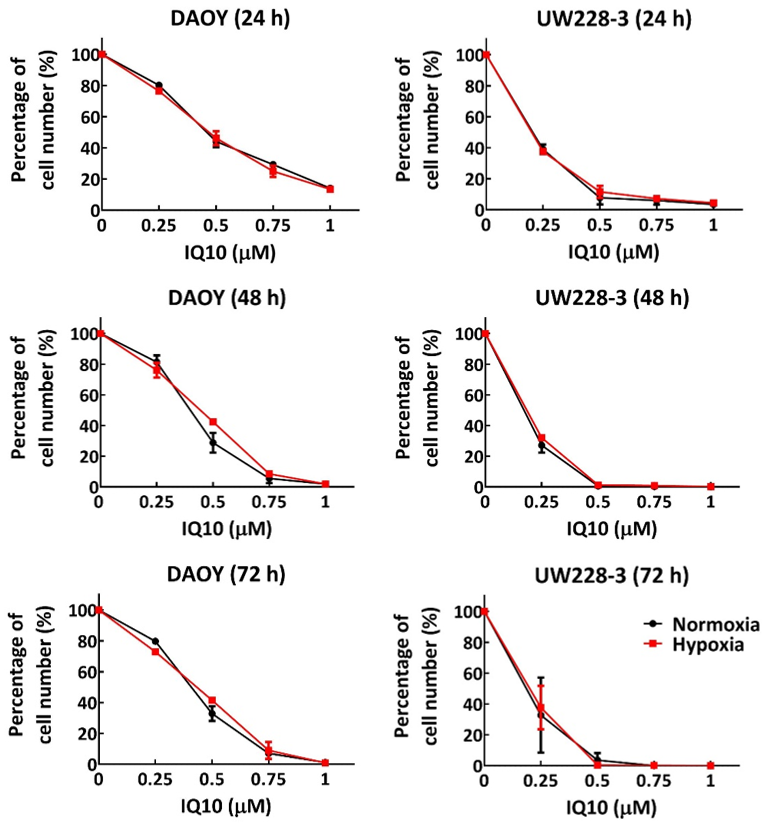


**Fig. S3 Radioresponse of brain cancer cells in normoxic *vs.* hypoxic conditions.** DAOY and UW228-3 cells were irradiated with single doses of 2, 4, 6 or 8 Gy under normoxic or hypoxic conditions (sham irradiated cells (0 Gy) were set as controls). Surviving fraction, from clonogenic survival assays, is plotted as a function of dose. Plating efficiencies for DAOY and UW228-3 cells cultured in normoxia were 64% and 91% (±11% and 5%), respectively; Plating efficiencies in hypoxia were 62% and 87% (±10% and 6%), respectively. Data represent the mean ± SD of at least three independent experiments, with each experiment containing six parallel data sets. ***P*< 0.01 and ****P*< 0.001 (Student’s *t*-test).


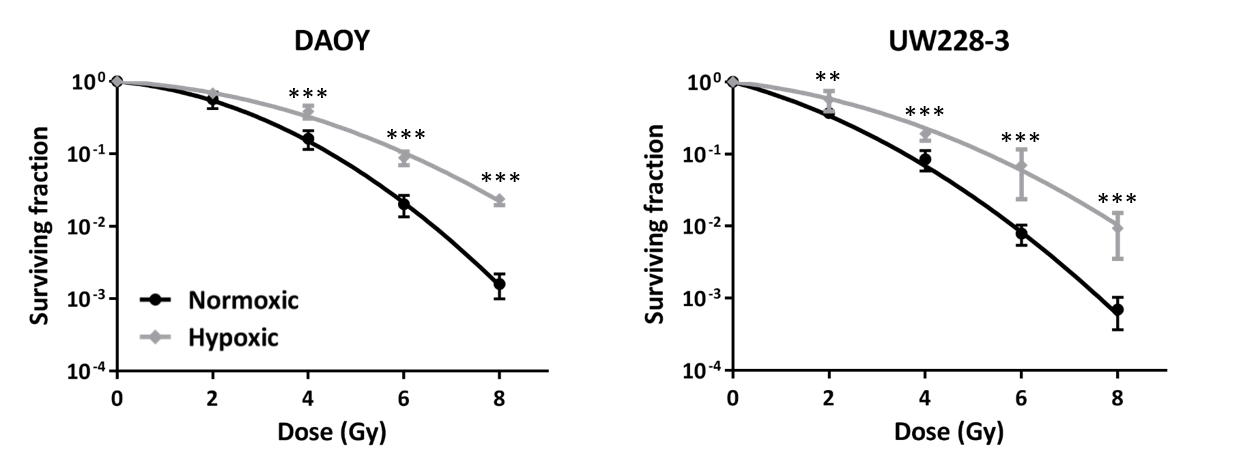


**Fig. S4 Representative images of γH2AX immunofluorescent staining in DAOY and UW228-3 cells.** Cells were treated with or without IQ10 (1 µM) for 4 h prior to irradiation (2 Gy). DNA double-strand breaks were analysed by confocal microscopy using γH2AX foci measurements. Red, γH2AX; Blue, DAPI fluorescent stain. Scale bar = 40 μm. Abbreviation: IR, irradiation.


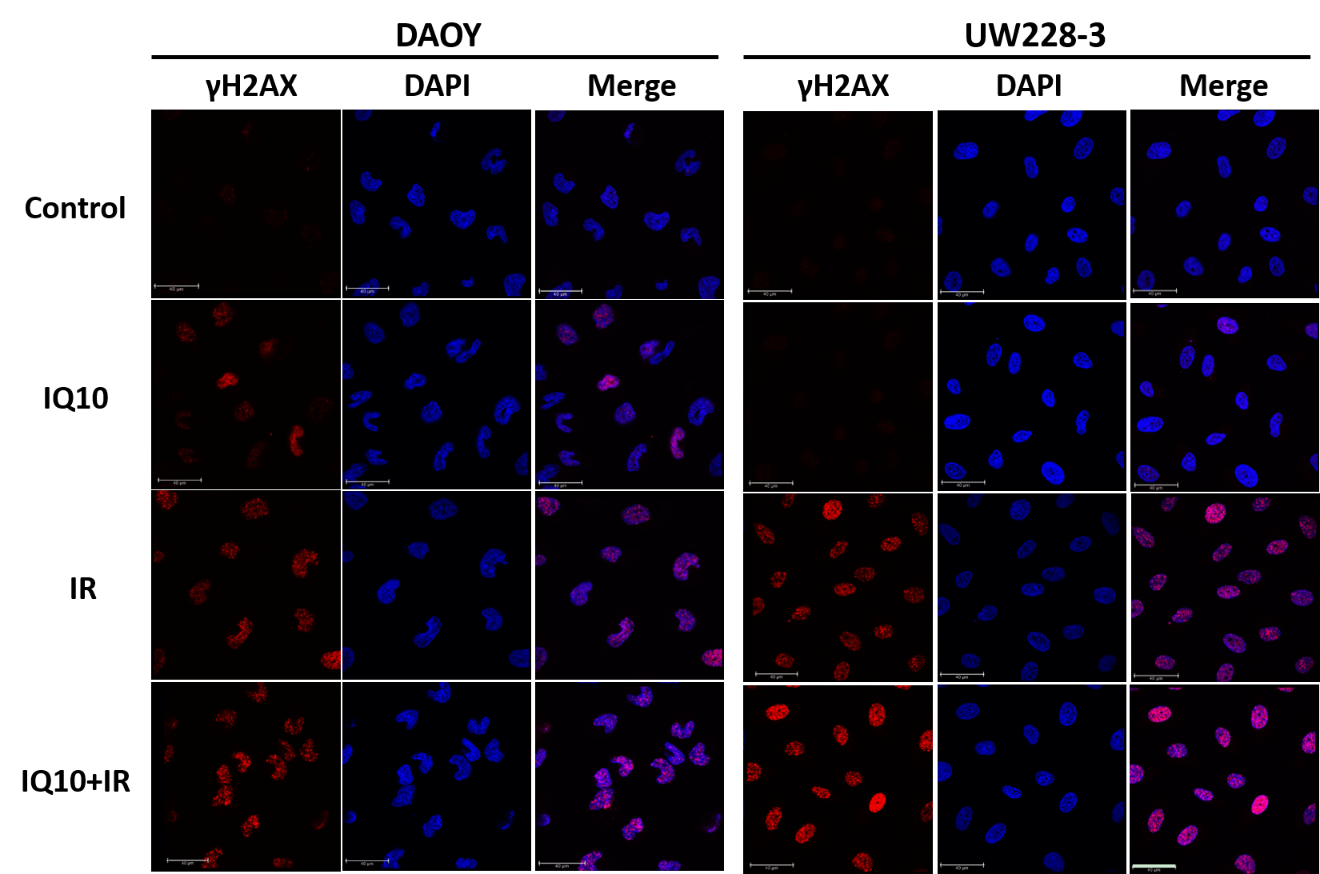


**Fig. S5 Effect of IQ10 on TrxR activity after 4 or 48 h of treatment in brain cancer cells.** Cells were treated with various concentrations of IQ10 for 4 or 48 h. TrxR activity was measured using the endpoint insulin reduction assay. (A) Inhibition of TrxR activity in SF188, DAOY and UW228-3 cells by IQ10 after 4 h of treatment. (B) Inhibition of TrxR activity in SF188 and DAOY cells after 48 h of treatment. Data are expressed as a percentage of DMSO-treated control. Data represent the mean ± SD of three independent experiments, with each conducted in duplicate. Blue represents SF188, black represents DAOY, green represents UW228-3.


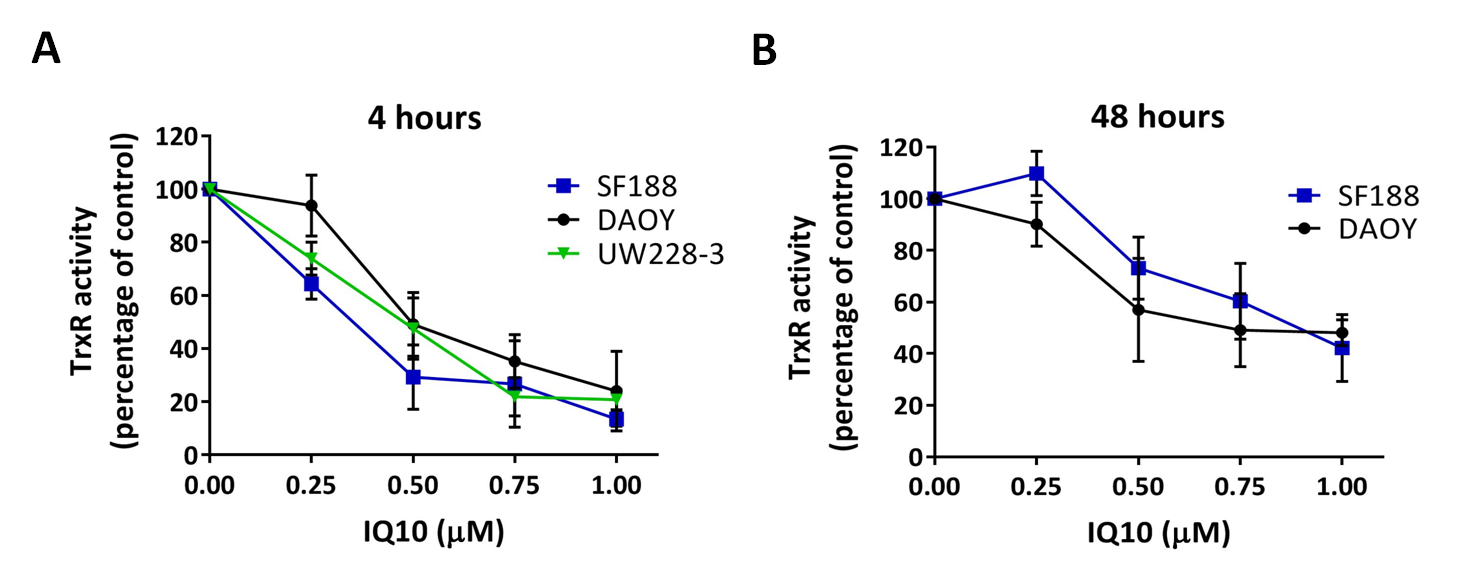


Table S1 The clonogenic assay IC_50_ values (μM) of TMZ and IQ10 in various cell lines at 48 h post-treatment.

| **Cell lines** | **TMZ** | **IQ10** |
| --- | --- | --- |
| SNB19 | 63.7±10.0 | >1 |
| DAOY | 463.5±20.1 | 0.4±0.1 |
| UW228-3 | 93.1±30.0 | 0.3±0.0 |
| SF188 | 15.2±0.9 | 0.3±0.1 |
| KNS42 | 49.5±11.4 | 1.0±0.2 |
| MRC5 | NA | >1 |

Data are expressed as mean ± SD of three independent experiments,with each experiment containing six parallel data sets. Abbreviations: TMZ, temozolomide; NA, not applicable/not performed.

Table S2 Comparison of IC_50_ values (μM) of IQ10 between normoxia and hypoxia in DAOY and UW228-3 cells.

| **Assays** | **Cell lines** | **Normoxia** | | | **Hypoxia** | | |
| --- | --- | --- | --- | --- | --- | --- | --- |
|  |  | **24 h** | **48 h** | **72 h** | **24 h** | **48 h** | **72 h** |
| Growth curve | DAOY | 0.47±0.01 | 0.38±0.02 | 0.38±0.01 | 0.45±0.06 | 0.39±0.03 | 0.38±0.02 |
|  | UW228-3 | 0.28±0.02 | 0.22±0.01 | 0.21±0.06 | 0.22±0.06 | 0.21±0.01 | 0.22±0.06 |
| Clonogenic survival | DAOY | NA | 0.40±0.09 | NA | NA | 0.41±0.13 | NA |
|  | UW228-3 | NA | 0.25±0.00 | NA | NA | 0.26±0.04 | NA |

Data are expressed as mean ± SD of three independent experiments, with each growth curve experiment performed in triplicate and each clonogenic survival experiment conducted in six parallel data sets. The clonogenic survival assays were only performed at the 48 h time point. NA, not applicable.

Table S3 Resistance profile toward IQ10 of 3D spheroids in comparison to 2D monolayer cells.

| **Cell lines** | **IC_50_ (µM)** | | **Degree of resistance** |
| --- | --- | --- | --- |
|  | **2D monolayers** | **3D spheroids** |  |
| Resazurin assay |  |  |  |
| DAOY | 0.29±0.08 | 0.64±0.10 | 2.21 |
| UW228-3 | 0.16±0.04 | >1 | >6.25 |
| Clonogenic assay |  |  |  |
| DAOY | 0.37±0.03 | 0.56±0.07 | 1.51 |
| UW228-3 | 0.30±0.04 | 0.83±0.09 | 2.77 |

Data are expressed as mean ± SD of three independent experiments, with each resazurin assay performed in triplicate and each clonogenic survival experiment conducted in six parallel data sets. The degree of resistance was calculated as the ratio of IC_50_ values of 3D spheroids and 2D monolayer cells.

Table S4 Radiobiologic parameters of brain cancer spheroids treated with IQ10 and/or radiation.

| **Spheroids** | **α (Gy^-1^)** | **β (Gy^-2^)** | **α/β (Gy)** | **SF2** | **SER_0.01_** |
| --- | --- | --- | --- | --- | --- |
| DAOY |  |  |  |  |  |
| IR alone | 0.08±0.06 | 0.13±0.03 | 0.66±0.55 | 0.55±0.04 | 1.38±0.31 |
| IR+IQ10 | 0.68±0.62 | 0.10±0.06 | 14.26±19.46 | 0.23±0.17 |  |
| *t* test | *P*=0.100 | *P*=0.421 | *P*=0.208 | ***P*=0.013** |  |
| UW228-3 |  |  |  |  |  |
| IR alone | 0.17±0.15 | 0.20±0.03 | 0.90±0.82 | 0.33±0.07 | 1.16±0.01 |
| IR+IQ10 | 0.28±0.30 | 0.24±0.06 | 1.41±1.74 | 0.23±0.08 |  |
| *t* test | *P*=0.599 | *P*=0.303 | *P*=0.669 | *P*=0.166 |  |

Parameters were calculated from clonogenic data fitted to the linear-quadratic model. Data represent the mean ± SD of at least three independent experiments, with each containing six parallel data sets. Statistical significance was determined by Student's *t*-test.The *P*-values are bold where they are ≤ 0.05. Abbreviations: IR, irradiation; SER_0.01_, sensitiser enhancement ratio at 1% survival; SF2, surviving fraction at 2 Gy.

Table S5 IC_50_ values of IQ10 in TrxR siRNA-treated *vs.* normal control cells.

| **Cell lines** | **IC_50_ (µM)** | | | |
| --- | --- | --- | --- | --- |
|  | **Growth curve** | **Fold-change** | **Clonogenic** | **Fold-change** |
| DAOY |  |  |  |  |
| control | 0.49 ± 0.07 | 1.53 | 0.36 ± 0.02 | 1.69 |
| siRNA | 0.75 ± 0.08 |  | 0.61 ± 0.06 |  |
| SF188 |  |  |  |  |
| control | 0.22 ± 0.04 | 1.73 | 0.31 ± 0.03 | 2.19 |
| siRNA | 0.38 ± 0.04 |  | 0.68 ± 0.08 |  |

Data are expressed as mean ± SD of three independent experiments, with each growth curve experiment performed in triplicate and each clonogenic survival experiment conducted in six parallel data sets. The fold-change was calculated as the ratio of IC_50_ values of TrxRsiRNA treated and normal control cells.

Table S6 Profiler PCR array results (vehicle control UW228-3 cells *vs.* DAOY cells).

| **Gene** | **Description** | **Fold-change** | | | |
| --- | --- | --- | --- | --- | --- |
|  |  | **4h** | | **24h** | |
| BMP7 | Bone morphogenetic protein 7 | -380.55 | A | -281.72 | A |
| CALD1 | Caldesmon 1 | -2.15 | \ | \ | \ |
| CAMK2N1 | Calcium/calmodulin-dependent protein kinase II inhibitor 1 | -13.52 | \ | -25.07 | A |
| CAV2 | Caveolin 2 | -7022.89 | A | -5153.72 | A |
| CDH1 | Cadherin 1, type 1, E-cadherin (epithelial) | 2.59 | B | \ | \ |
| CDH2 | Cadherin 2, type 1, N-cadherin (neuronal) | -2.22 | \ | \ | \ |
| COL1A2 | Collagen, type I, alpha 2 | \ | \ | 2.39 | \ |
| COL3A1 | Collagen, type III, alpha 1 | -2.03 | \ | \ | \ |
| COL5A2 | Collagen, type V, alpha 2 | 11.04 | \ | 11.6 | \ |
| CTNNB1 | Catenin (cadherin-associated protein), beta 1, 88kDa | -2.18 | \ | -3.35 | \ |
| DSC2 | Desmocollin 2 | -27.74 | A | -21.27 | A |
| DSP | Desmoplakin | -3290.25 | A | -1776.31 | A |
| EGFR | Epidermal growth factor receptor | -6.01 | \ | -4.41 | \ |
| ERBB3 | V-erb-b2erythroblastic leukemia viral oncogene homolog 3 (avian) | 2.69 | A | 13.09 | A |
| ESR1 | Estrogen receptor 1 | \ | \ | -3.3 | B |
| F11R | F11 receptor | \ | \ | 2.58 | \ |
| FGFBP1 | Fibroblast growth factor binding protein 1 | -4.26 | B | -10.77 | B |
| FN1 | Fibronectin 1 | -12.64 | \ | -15.22 | \ |
| FOXC2 | Forkhead box C2 (MFH-1, mesenchyme forkhead 1) | -1794.19 | A | -824.68 | A |
| FZD7 | Frizzled family receptor 7 | -4.41 | \ | -3.75 | \ |
| GNG11 | Guanine nucleotide binding protein (G protein), gamma 11 | -2.13 | \ | \ | \ |
| GSC | Goosecoidhomeobox | -156.79 | A | -196.15 | A |
| GSK3B | Glycogen synthase kinase 3 beta | -2.66 | \ | -2.27 | \ |
| IGFBP4 | Insulin-like growth factor binding protein 4 | -11539.35 | A | -7702.94 | A |
| IL1RN | Interleukin 1 receptor antagonist | -95.04 | A | -15.92 | A |
| ILK | Integrin-linked kinase | -2.49 | \ | \ | \ |
| ITGA5 | Integrin, alpha 5 (fibronectin receptor, alpha polypeptide) | 3.51 | \ | 3.85 | \ |
| KRT14 | Keratin 14 | -11.10 | A | -6.21 | A |
| KRT19 | Keratin 19 | 20.94 | A | 8.3 | B |
| KRT7 | Keratin 7 | \ | \ | -2.67 | B |
| MMP2 | Matrix metallopeptidase 2 (gelatinase A, 72kDagelatinase, 72kDa type IV collagenase) | -20.86 | \ | -15.57 | \ |
| MMP3 | Matrix metallopeptidase 3 (stromelysin 1, progelatinase) | 167.11 | A | 57.41 | A |

**Table S6 (continued).**

| MMP9 | Matrix metallopeptidase 9 (gelatinase B, 92kDagelatinase, 92kDa type IV collagenase) | -51.41 | A | -55.45 | A |
| --- | --- | --- | --- | --- | --- |
| MST1R | Macrophage stimulating 1 receptor (c-met-related tyrosine kinase) | 2.96 | B | -16.34 | A |
| NODAL | Nodal homolog (mouse) | -27.81 | B | -19.77 | B |
| NOTCH1 | Notch 1 | \ | \ | 2.85 | A |
| NUDT13 | Nudix (nucleoside diphosphate linked moiety X)-type motif 13 | -5.86 | A | -4.26 | A |
| OCLN | Occludin | -11.90 |  | -8.10 | \ |
| PDGFRB | Platelet-derived growth factor receptor, beta polypeptide | -13.37 | B | -13.65 | B |
| PLEK2 | Pleckstrin 2 | -88.98 | A | -269.41 | A |
| PTK2 | PTK2 protein tyrosine kinase 2 | -3.01 | \ | -2.95 | \ |
| SERPINE1 | Serpin peptidase inhibitor, clade E (nexin, plasminogen activator inhibitor type 1), member 1 | -3.57 | \ | -8.62 | \ |
| GEMIN2 | Survival of motor neuron protein interacting protein 1 | -2.45 | \ | -2.52 | \ |
| SNAI1 | Snail homolog 1 (Drosophila) | -79.23 | A | -72.45 | A |
| SNAI2 | Snail homolog 2 (Drosophila) | -4.33 | \ | -4.49 | \ |
| SNAI3 | Snail homolog 3 (Drosophila) | -6.62 | B | -11.02 | B |
| SPARC | Secreted protein, acidic, cysteine-rich (osteonectin) | 2.21 | \ | 2.67 | \ |
| SPP1 | Secreted phosphoprotein 1 | 61.01 | \ | 44.92 | \ |
| STEAP1 | Six transmembrane epithelial antigen of the prostate 1 | -2.84 | \ | -2.28 | \ |
| TFPI2 | Tissue factor pathway inhibitor 2 | -99.82 | \ | -134.92 | \ |
| TGFB2 | Transforming growth factor, beta 2 | 8.44 | \ | 6.52 | \ |
| TGFB3 | Transforming growth factor, beta 3 | 5.50 | \ | 6.07 | \ |
| TMEFF1 | Transmembrane protein with EGF-like and two follistatin-like domains 1 | -6.24 | \ | -4.63 | \ |
| TMEM132A | Transmembrane protein 132A | -4.43 | \ | -3.42 | \ |
| TSPAN13 | Tetraspanin 13 | -4.23 | \ | -2.79 | \ |
| TWIST1 | Twist homolog 1 (Drosophila) | -6.22 | \ | -4.78 | \ |
| VCAN | Versican | 2.65 | \ | 2.65 | \ |
| VIM | Vimentin | -2.02 | \ | \ | \ |
| WNT5A | Wingless-type MMTV integration site family, member 5A | -32.64 | \ | -26.38 | \ |
| ZEB2 | Zinc finger E-box binding homeobox 2 | -2.14 | \ | \ | \ |

A: This gene’s expression is relatively low (threshold cycle> 30) in one sample and reasonably detected (threshold cycle < 30) in the other sample suggesting that the actual fold-change value is at least as large as the calculated and reported fold-change result.

B: This gene’s average relative expression level is low (threshold cycle> 30) in both control and test samples.

Table S7 Profiler PCR array results for DAOY and UW228-3 cells (IQ10-treated group *vs.* vehicle control group at two time points).

| **Gene** | **Description** | **Fold-change** | |
| --- | --- | --- | --- |
| **Up-regulated gene expression in DAOY** | | **4h** | **24h** |
| ERBB3 | V-erb-b2erythroblastic leukemia viral oncogene homolog 3 (avian) | \ | 5.94 (B) |
| GNG11 | Guanine nucleotide binding protein (G protein), gamma 11 | \ | 2.16 |
| JAG1 | Jagged 1 | \ | 2.85 |
| KRT19 | Keratin 19 | 9.49 | \ |
| SNAI1 | Snail homolog 1 (Drosophila) | 2.37 | \ |
| **Up-regulated gene expression in UW228-3** | | **4h** | **24h** |
| BMP2 | Bone morphogenetic protein 2 | \ | 5.43 (A) |
| CAMK2N1 | Calcium/calmodulin-dependent protein kinase II inhibitor 1 | \ | 4.56 (A) |
| DSP | Desmoplakin | 2.11 (B) | \ |
| ESR1 | Estrogen receptor 1 | 2.55(B) | \ |
| FOXC2 | Forkhead box C2 (MFH-1, mesenchyme forkhead 1) | 3.39 (B) | \ |
| IGFBP4 | Insulin-like growth factor binding protein 4 | 3.34 (B) | \ |
| JAG1 | Jagged 1 | \ | 2.31 |
| KRT14 | Keratin 14 | 2.23 (B) | \ |
| MMP3 | Matrix metallopeptidase 3 (stromelysin 1, progelatinase) | \ | 2.23 |
| PLEK2 | Pleckstrin 2 | \ | 7.01 |
| RGS2 | Regulator of G-protein signalling 2, 24kDa | 2.20 | \ |
| SNAI2 | Snail homolog 2 (Drosophila) | 2.87 | \ |
| SNAI3 | Snail homolog 3 (Drosophila) | 4.72 (B) | \ |
| **Down-regulated gene expression in DAOY** | | **4h** | **24h** |
| COL1A2 | Collagen, type I, alpha 2 | \ | -2.79 |
| COL3A1 | Collagen, type III, alpha 1 | \ | -2.06 |
| DSP | Desmoplakin | -2.05 | \ |
| EGFR | Epidermal growth factor receptor | -2.34 | \ |
| ERBB3 | V-erb-b2erythroblastic leukemia viral oncogene homolog 3 (avian) | -2.98 (B) | \ |
| ESR1 | Estrogen receptor 1 | \ | -2.69 (B) |
| FGFBP1 | Fibroblast growth factor binding protein 1 | -3.85 (B) | -3.46 (B) |
| FN1 | Fibronectin 1 | \ | -2.23 |
| FOXC2 | Forkhead box C2 (MFH-1, mesenchyme forkhead 1) | \ | -2.39 |
| FZD7 | Frizzled family receptor 7 | \ | -2.19 |
| GSK3B | Glycogen synthase kinase 3 beta | -2.27 | -2.00 |
| IL1RN | Interleukin 1 receptor antagonist | -2.01 | -6.02 (A) |
| ITGA5 | Integrin, alpha 5 (fibronectin receptor, alpha polypeptide) | -2.02 | \ |
| ITGB1 | Integrin, beta 1 (fibronectin receptor, beta polypeptide, antigen CD29 includes MDF2, MSK12) | \ | -2.51 |
| KRT7 | Keratin 7 | \ | -9.25 (B) |

**Table S7 (continued).**

| MAP1B | Microtubule-associated protein 1B | -2.13 | \ |
| --- | --- | --- | --- |
| MST1R | Macrophage stimulating 1 receptor (c-met-related tyrosine kinase) | \ | -69.07 (A) |
| NOTCH1 | Notch 1 | -2.40 (A) | \ |
| NUDT13 | Nudix (nucleoside diphosphate linked moiety X)-type motif 13 | \ | -11.39 (A) |
| PDGFRB | Platelet-derived growth factor receptor, beta polypeptide | \ | -8.17 (B) |
| PTK2 | PTK2 protein tyrosine kinase 2 | -2.23 | \ |
| SERPINE1 | Serpin peptidase inhibitor, clade E (nexin, plasminogen activator inhibitor type 1), member 1 | \ | -3.46 |
| SNAI3 | Snail homolog 3 (Drosophila) | -5.75 (B) | \ |
| TCF4 | Transcription factor 4 | -4.27 | \ |
| TGFB2 | Transforming growth factor, beta 2 | -4.21 | \ |
| TWIST1 | Twist homolog 1 (Drosophila) | -2.02 | \ |
| VPS13A | Vacuolar protein sorting 13 homolog A (S. cerevisiae) | \ | -2.87 |
| WNT5A | Wingless-type MMTV integration site family, member 5A | -2.3 | -2.25 |
| WNT5B | Wingless-type MMTV integration site family, member 5B | -5.52 | \ |
| ZEB1 | Zinc finger E-box binding homeobox 1 | -2.40 | \ |
| ZEB2 | Zinc finger E-box binding homeobox 2 | -2.70 | \ |
| **Down-regulated gene expression in UW228-3** | | **4h** | **24h** |
| AKT1 | V-akt murine thymoma viral oncogene homolog 1 | \ | -2.22 |
| CALD1 | Caldesmon 1 | \ | -5.50 |
| CDH1 | Cadherin 1, type 1, E-cadherin (epithelial) | -3.07 (B) | \ |
| CDH2 | Cadherin 2, type 1, N-cadherin (neuronal) | \ | -3.73 |
| COL1A2 | Collagen, type I, alpha 2 | \ | -8.40 |
| COL3A1 | Collagen, type III, alpha 1 | \ | -3.03 |
| COL5A2 | Collagen, type V, alpha 2 | \ | -5.50 |
| CTNNB1 | Catenin (cadherin-associated protein), beta 1, 88kDa | \ | -2.13 |
| DSC2 | Desmocollin 2 | \ | -9.71 (B) |
| ERBB3 | V-erb-b2erythroblastic leukemia viral oncogene homolog 3 (avian) | -2.50 (A) | -2.33 (A) |
| F11R | F11 receptor | \ | -3.84 |
| FZD7 | Frizzled family receptor 7 | -2.17 | -2.95 |
| GEMIN2 | Survival of motor neuron protein interacting protein 1 | \ | -2.43 |
| GSK3B | Glycogen synthase kinase 3 beta | -2.17 | -2.55 |
| IL1RN | Interleukin 1 receptor antagonist | \ | -2.43 |
| ILK | Integrin-linked kinase | \ | -2.39 |
| ITGA5 | Integrin, alpha 5 (fibronectin receptor, alpha polypeptide) | \ | -2.35 |
| ITGAV | Integrin, alpha V (vitronectin receptor, alpha polypeptide, antigen CD51) | \ | -3.07 |
| ITGB1 | Integrin, beta 1 (fibronectin receptor, beta polypeptide, antigen CD29 includes MDF2, MSK12) | \ | -2.75 |

**Table S7 (continued).**

| KRT19 | Keratin 19 | -4.38 (A) | -18.77 (B) |
| --- | --- | --- | --- |
| MAP1B | Microtubule-associated protein 1B | -2.55 | -2.22 |
| MSN | Moesin | \ | -2.46 |
| MST1R | Macrophage stimulating 1 receptor (c-met-related tyrosine kinase) | -3.51 (B) | \ |
| NOTCH1 | Notch 1 | -4.32 (A) | -6.68 (A) |
| NUDT13 | Nudix (nucleoside diphosphate linked moiety X)-type motif 13 | \ | -2.85 |
| OCLN | Occludin | \ | -7.62 |
| PTK2 | PTK2 protein tyrosine kinase 2 | -2.08 | \ |
| RAC1 | Ras-related C3botulinum toxin substrate 1 (rho family, small GTP binding protein Rac1) | \ | -2.57 |
| SMAD2 | SMAD family member 2 | \ | -2.19 |
| SPARC | Secreted protein, acidic, cysteine-rich (osteonectin) | \ | -2.08 |
| STAT3 | Signal transducer and activator of transcription 3 (acute-phase response factor) | \ | -2.81 |
| TCF3 | Transcription factor 3 (E2A immunoglobulin enhancer binding factors E12/E47) | \ | -3.07 |
| TCF4 | Transcription factor 4 | -2.16 | -2.19 |
| TGFB1 | Transforming growth factor, beta 1 | \ | -2.14 |
| TGFB2 | Transforming growth factor, beta 2 | \ | -2.97 |
| TGFB3 | Transforming growth factor, beta 3 | -2.10 | -8.57 |
| TMEFF1 | Transmembrane protein with EGF-like and two follistatin-like domains 1 | \ | -4.96 |
| TWIST1 | Twist homolog 1 (Drosophila) | -4.29 | -2.45 |
| VIM | Vimentin | \ | -2.14 |
| VPS13A | Vacuolar protein sorting 13 homolog A (S. cerevisiae) | \ | -2.51 |
| WNT5A | Wingless-type MMTV integration site family, member 5A | -2.89 | \ |
| WNT5B | Wingless-type MMTV integration site family, member 5B | -4.00 | -4.92 |
| ZEB2 | Zinc finger E-box binding homeobox 2 | \ | -2.68 |

A: This gene’s expression is relatively low (threshold cycle> 30) in one sample and reasonably detected (threshold cycle < 30) in the other sample suggesting that the actual fold-change value is at least as large as the calculated and reported fold-change result.

B: This gene’s average relative expression level is low (threshold cycle> 30) in both control and test samples.

**Table S8 Cell culture media formulations.**

| **Cell Lines** | **Reagents** | **Source** | **Volume (mL)** |
| --- | --- | --- | --- |
| **SNB19 DAOY** | Minimum essential medium eagle (MEM) (with Earle’s salts and sodium bicarbonate, without L-glutamine) | M2279,Sigma | 500 |
|  | Heatinactivatedironsupplementeddonorbovine serum | 10371029, Gibco, Life Technologies | 58 |
|  | Penicillin/Streptomycin(10,000unitspenicillinand10mg streptomycin/mL) | P0781,Sigma | 5.8 |
|  | L-Glutamine(200mM) | G7513,Sigma | 5.8 |
|  | MEM non-essentialaminoacidssolution(100x) | M7145,Sigma | 5.8 |
|  | Sodium pyruvate solution (100mM) | S8636, Sigma | 5.8 |
| **SF188 KNS42** | Dulbecco's modified eagle medium: nutrient mixture F-12 (DMEM/F-12) | 21331-020, Gibco, Life Technologies | 500 |
|  | Heatinactivatedironsupplementeddonorbovine serum | 10371029, Gibco, Life Technologies | 57 |
|  | Penicillin/Streptomycin (10,000 units penicillin and 10 mg streptomycin/mL) | P0781, Sigma | 5.7 |
|  | L-Glutamine (200 mM) | G7513, Sigma | 5.7 |
| **UW228-3** | Dulbecco’s modified eagle’s medium/Nutrient Mixture F-12 Ham | D6421,Sigma | 500 |
|  | Heatinactivatedironsupplementeddonorbovine serum | 10371029, Gibco, Life Technologies | 91.5 |
|  | Penicillin/Streptomycin(10,000unitspenicillinand10mg streptomycin/mL) | G7513,Sigma | 6.1 |
|  | L-Glutamine (200 mM) | S8636, Sigma | 6.1 |
|  | Sodium pyruvate (100 mM) | P0781,Sigma | 6.1 |
| **MRC5** | Minimum essential medium eagle (MEM) (with Earle’s salts and sodium bicarbonate, without L-glutamine) | M2279, Sigma | 500 |
|  | Heat inactivated iron supplemented donor bovine serum | 10371029, Gibco, Life Technologies | 59 |
|  | Penicillin/Streptomycin (10,000 units penicillin and 10 mg streptomycin/mL) | P0781, Sigma | 5.9 |
|  | L-glutamine (200 mM) | G7513, Sigma | 5.9 |
|  | MEM non-essential amino acids solution (100x) | M7145, Sigma | 5.9 |
|  | Hepes solution (1 M) | H0887, Sigma | 5.9 |
|  | Sodium carbonate solution (7.5%) | S8761, Sigma | 5.9 |
